# Assessing analytical convolution effects in diffusion studies: Applications to experimental and natural diffusion profiles

**DOI:** 10.1371/journal.pone.0241788

**Published:** 2020-11-24

**Authors:** Michael C. Jollands

**Affiliations:** 1 Institute of Earth Sciences, University of Lausanne, Lausanne, Switzerland; 2 Lamont-Doherty Earth Observatory, Palisades, New York, United States of America; University of Genova, ITALY

## Abstract

Given that all in-situ analytical techniques have a non-zero beam size, all measured profiles, resulting from diffusion or otherwise, will be artefactually elongated to some degree. Profiles where the total length over which the concentration changes approaches the resolution of the analytical technique likely suffer from serious convolution; the measured profiles may be considerably elongated relative to the true profile. Resolving this effect is non-trivial, except for some specific combinations of profile type and beam geometry. In this study, a versatile method for numerically deconvoluting diffusion profiles acquired using techniques with Gaussian, Lorentzian, (pseudo-)Voigt, circular/elliptical or square/rectangular interaction volumes, is presented. A MATLAB code, including a user-friendly interface (PACE-the Program for Assessing Convolution Effects in diffusion studies), is also provided, and applied to several experimental and natural profiles interpreted as resulting from diffusion, showing various degrees of convolution.

## Introduction

Diffusion modelling is now commonplace in the earth sciences, given its ability to determine timescales of geological processes regardless of their absolute age [1–5]. Currently, the literature contains some open questions regarding discrepancies between different diffusion coefficients and the applicability of diffusion coefficients determined in the laboratory to natural systems. However, the effects of analytical convolution, normally artificial broadening of diffusion profiles, can, in some cases, be assessed using various analytical and numerical approaches [6–11].

**Fig 1:**
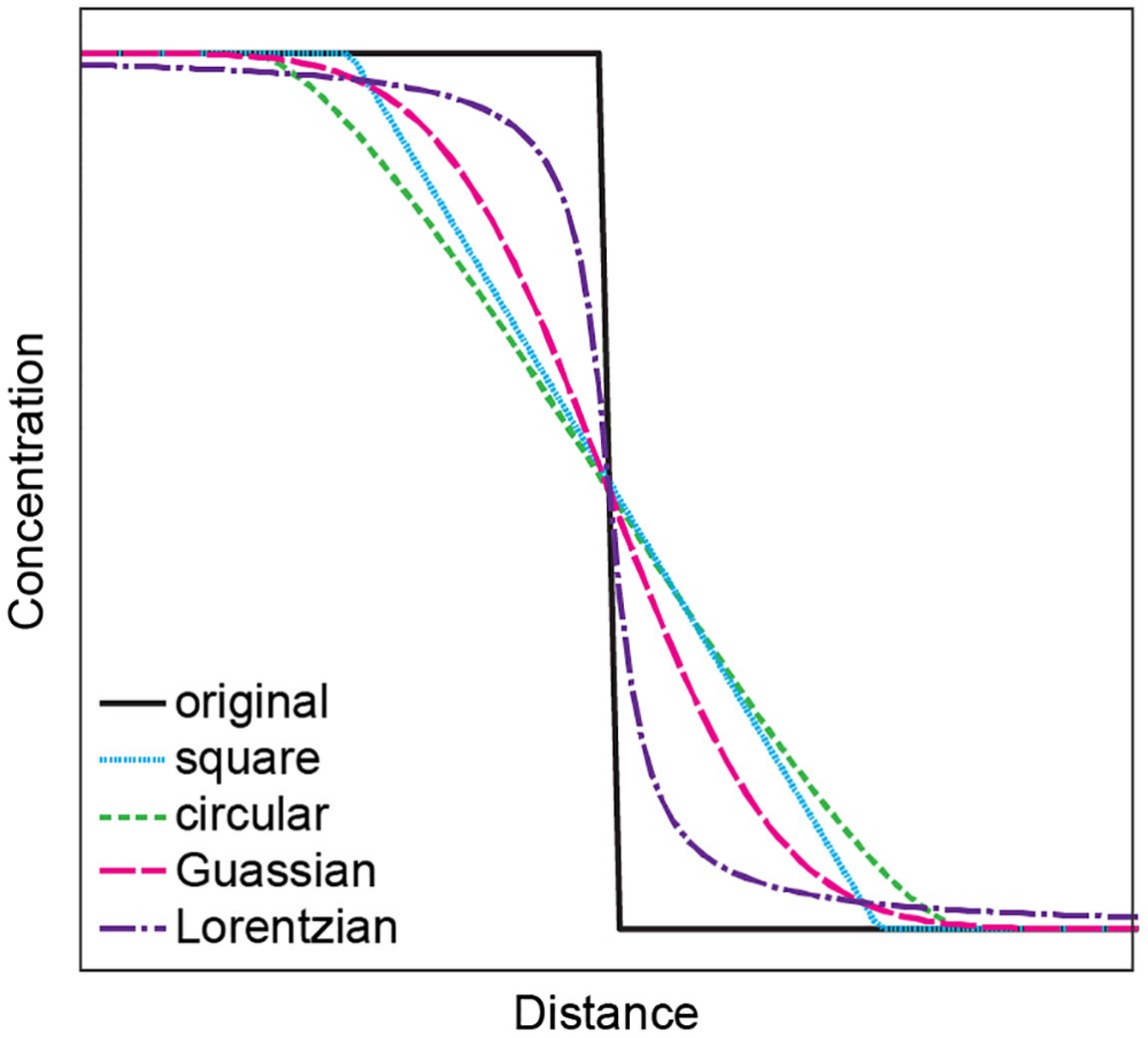
Types of convolution considered in this study. The pseudo-Voigt profile (not shown) is a linear combination of Gaussian and Lorentzian profiles.

The error function, which is fundamental to several analytical solutions of Fick’s second law, has a geometry that corresponds to the cumulative distribution function of the normal distribution. This means that analysing a simple step function, using an analytical method where the interaction volume can be described as Gaussian, will yield an error function. Likewise, analysing an error function-shaped profile with any Gaussian beam (e.g. most analyses using nanoSIMS, electron probe, scanning electron microscope, cathodoluminescence, Fourier transform infrared spectroscopy) will lead to some smoothing-out/lengthening of the error function, whilst maintaining its form. The effect of beam convolution, in the simple case a Gaussian beam, has been derived both by Ganguly et al. and Arnould and Hild [7, 12] as:

$${\text{D}}^{*}{\text{t}=\text{Dt}+\sigma }^{2}/2$$ (1)

where D* is the measured diffusion coefficient, D is the actual diffusion coefficient, σ is the standard deviation associated with a beam with a Gaussian interaction volume and t is the time for diffusion. The characteristic length of a diffusion profile is proportional to √(Dt). From (1), the effect of convolution ((D*t)/(Dt)) is, intuitively, greatest for profiles with high σ and low Dt. Circular/elliptical, square/rectangular (e.g. laser ablation inductively coupled plasma mass spectrometry, LA-ICP-MS) and Lorentzian/(pseudo-)Voigt (some electron probe analyses) beams will also convolute profiles, with different resulting shapes (Fig 1).

**Fig 2:**
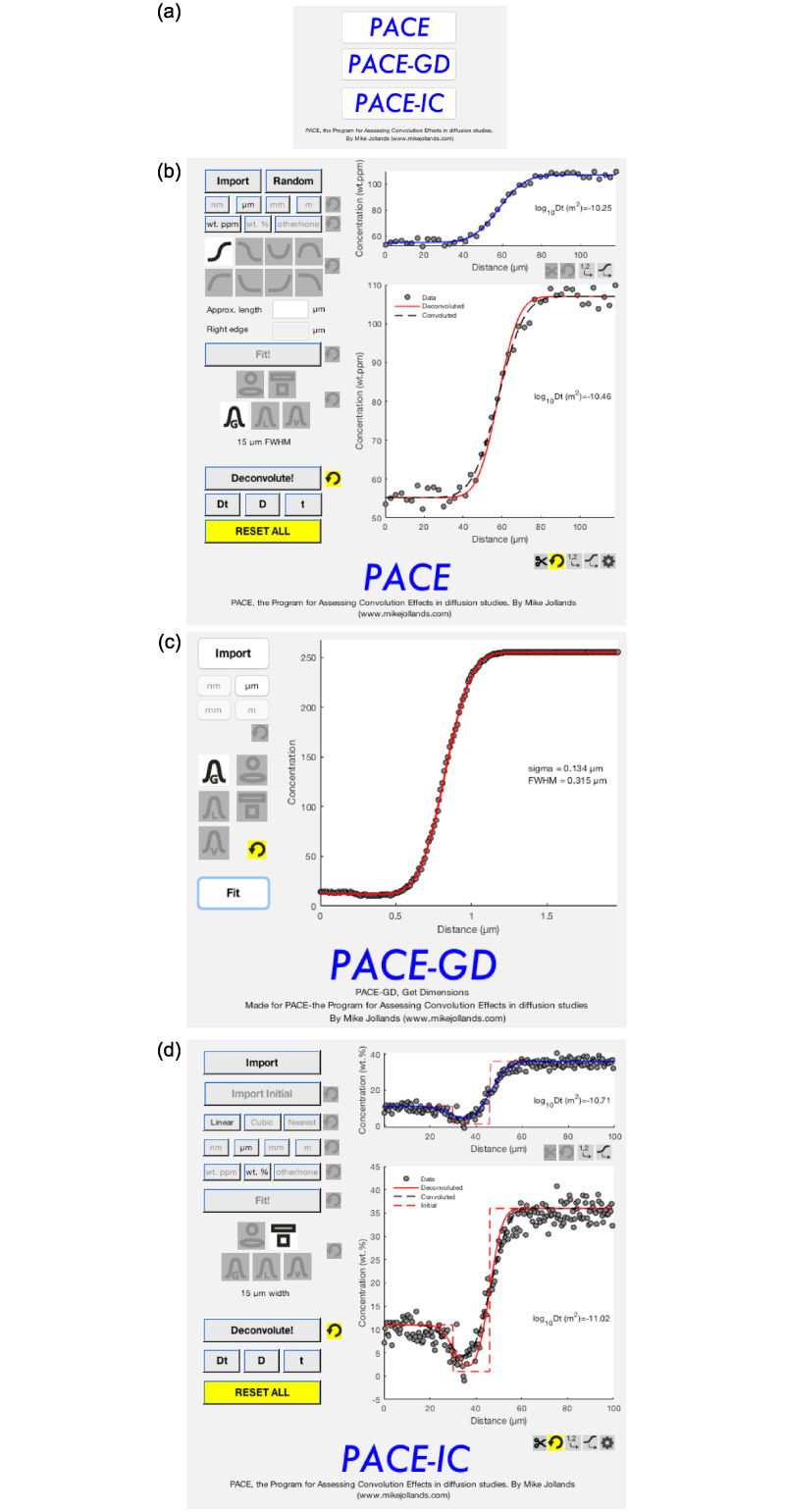
Screenshots from PACE, PACE-GD and PACE-IC. (a) shows the start screen, from which the others are accessed. (b) is the main screen from PACE, following fitting and deconvolution of a random profile. The top right hand panel shows the initial fit (blue line) to the data (points), then the lower panel shows the deconvoluted (solid red line) and convoluted (dashed black line) fits, incorporating a Gaussian beam with a 15 μm full width at half-maximum (c) shows PACE-GD following extraction of a beam size from a Gaussian convoluted profile. (d) shows PACE-IC following fitting and deconvolution of a profile with a stepped initial condition, and a 15 μm wide square/rectangular beam. The top panel shows the initial fit using the imported initial condition (red dashed line), then the deconvoluted profile, as in (b).

Whilst this convolution effect has been recognised for several decades, it is still often omitted from diffusion studies. Therefore, to enable the fast, reproducible assessment of convolution effects, PACE (the Program for Assessing Convolution Effects in diffusion studies) is presented (Fig 2). PACE is a stand-alone package (for Mac OSX and Microsoft Windows), built in MATLAB, allowing extraction of deconvoluted profiles from measured profiles where 1) circular, square, Gaussian, Lorentzian or pseudo-Voigt interaction volumes can be assumed; 2) where the beam size is known *a priori*, or can be estimated and 3) where an assumption can be made regarding the true nature, i.e. the geometry, of the profile. A second program- PACE-IC (PACE-Initial Conditions) is also provided, this is based on PACE, but allows the user to input initial and boundary conditions. In addition, a third program—PACE-GD (Get Dimensions) is provided for estimating beam sizes from profiles made across known step functions, with any of the interaction types listed above.

The software does not assume knowledge of programming or diffusion past simple recognition of profile types. The use of PACE is demonstrated on several diffusion profiles with different beam geometries, showing both cases where the convolution effects are negligible, and where they may become important.

## Methods

### Solutions to Fick’s second law (PACE)

The methodology used in PACE only employs analytical solutions to the diffusion equation—it does not incorporate any numerical diffusion modelling. Therefore, PACE can only be used in cases where the boundary conditions are fixed, and the initial conditions are describable using step functions. Numerical modelling is incorporated into PACE-IC (described below). This precludes the use of PACE in certain situations including 1) changing temperature, leading to partition/distribution coefficients changing over time and 2) loss of the original core composition following a prolonged period of diffusion.

For simulating sigmoidal shaped profiles that include flat plateaus on the left and right, eq (2) is used:

$$\text{C}(\text{x},\text{t})={\text{C}}_{2}+({\text{C}}_{1}{-\text{C}}_{2})\times \frac{1}{2}\times \text{erfc}(\frac{\text{x}-\text{X}}{2\sqrt{\text{Dt}}})$$ (2)

Where C(x,t) is the concentration at position x and time t, X is the midpoint/inflection point of the profile and D is the diffusion coefficient (m^2^s^-1^).

For U and upside-down-U shaped profiles, eq (3) is used:

$$\text{C}(\text{x},\text{t})={\text{C}}_{2}+({\text{C}}_{1}{-\text{C}}_{2})\times (\text{erfc}(\frac{\text{x}}{2\sqrt{\text{Dt}}})+\text{erfc}(\frac{\text{X-x}}{2\sqrt{\text{Dt}}}))$$ (3)

where in this case, C_1_ and C_2_ represent boundary and core concentrations, and X is the length of the system, i.e. the position of the right-hand boundary, where the left-hand boundary is at x = 0 by default.

For profiles where the boundary is at x = 0 (left hand side) and the system is considered semi-infinite, eq (4) is used:

$$\text{C}(\text{x},\text{t})={\text{C}}_{2}+({\text{C}}_{1}{-\text{C}}_{2})\times \text{erfc}(\frac{\text{x}}{2\sqrt{\text{Dt}}})$$ (4)

and where the boundary is on the right-hand side of the profile at x≠0, again in a semi-infinite system, eq (5) is used instead:

$$\text{C}(\text{x},\text{t})={\text{C}}_{2}+({\text{C}}_{1}{-\text{C}}_{2})\times \text{erfc}(\frac{\text{X-x}}{2\sqrt{\text{Dt}}})$$ (5)

where X is again the position of the boundary. By default, PACE outputs log_10_Dt, along with the respective concentrations. From this, D or t can be readily extracted when the other is known, or, because Dt = ∫D(t)dt [13], Dt can also be used to extract cooling histories.

### Numerical diffusion modelling (PACE-IC)

PACE-IC uses an explicit finite difference method to solve Fick’s second law numerically in one dimension. This method is described thoroughly elsewhere [14–1,6], but, briefly, the system is modelled as a vector representing a series of equidistant steps (spacing = Δx), with the initial and boundary conditions imported by the user. The model then steps forwards through time (size of time step = Δt), with the concentration (C) at each distance step (i) recalculated at every time step (j):

$${\text{C}}_{\text{i},\text{j}+1}{=\text{C}}_{\text{i},\text{j}}+D\Delta \text{t}(\frac{{\text{C}}_{\text{i}+1,\text{j}}{-2\text{C}}_{\text{i},\text{j}}{+\text{C}}_{\text{i}-1,\text{j}}}{{\Delta \text{x}}^{2}})$$ (6)

The size of the time step is defined by D and Δx, (DΔt)/Δx^2^ must be less than 0.5 for stability. PACE-IC assumes that the boundaries are constant over time.

### Simulating beam convolution

**Fig 3:**
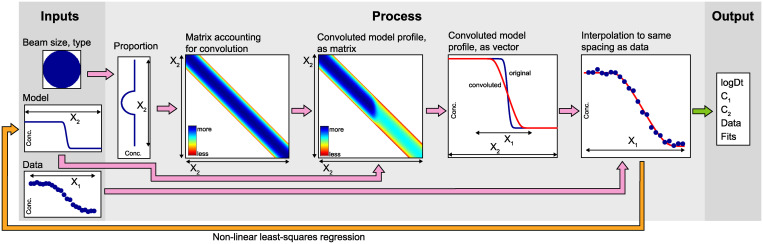
Method for convoluting diffusion profiles, described in the text.

A workflow describing how beam convolution is simulated in PACE is presented in Fig 3, with the methodology described herein.

The inputs are 1) details regarding the nature of the beam; 2) a model profile vector, which is generated from the non-linear least squares regression and 3) the measured profile, i.e. the data vector, which may or may not include uncertainties. The model profile vector is longer than the data vector, in that it extends past the highest and lowest distance values. This is shown in the ‘Inputs’ section of Fig 3 –the data vector has a total length X_1_, and the model profile vector has length X_2_, where X_2_>X_1_.

To simulate convolution, a square matrix is constructed (denoted ‘Matrix accounting for convolution’ in Fig 3), with dimensions X_2_ by X_2_. This is a matrix of zeros, with each column and row containing a vector representing the sampling density associated with a given beam type and size (shown as ‘Proportion’ in Fig 3). Notably, this calculation assumes uniform sampling density when using circular/elliptical and square/rectangular beams. For LA-ICP-MS analyses, this means flat-bottomed craters with vertical walls, which is a reasonable assumption for modern systems (e.g. Fig 1 of Neymark et al. [17]).

The matrix accounting for convolution is then multiplied by the model profile vector pointwise. This gives a new matrix, denoted ‘Convoluted model profile, as matrix’ in Fig 3.

This matrix is then summed along one dimension to form a vector, denoted ‘Convoluted model profile, as vector’ in Fig 3. The vector is then interpolated to find the nearest points to the data, and the difference between this vector and the data is minimised using the *lsqnonlin* solver in MATLAB. Whilst obviously slower computationally than the analytical solution for deconvoluting the effect of a Gaussian beam on an error function [7], this method is versatile—it can potentially be used for simulating any beam shape and any profile shape, as long as an assumption can be made regarding the nature of, i.e. the true form of, the deconvoluted profile.

### Treatment of boundaries

In order to simulate convolution, it is necessary to begin with a profile that is longer than the measured profile. In the case of the curves generated by eq (2), where the extremities of the curve are flat segments, the boundaries are simply extended. However, where the curve intersects a boundary with constant composition (e.g. eqs (3)–(5)), the situation is slightly more complex. In PACE, the boundaries in these equations are considered as fixed. This means that the convolution effect becomes smaller towards the boundary, i.e., in the most extreme case, a point made exactly at the boundary using a Gaussian beam will only be convoluted by the inside of the crystal, and not the outside. Whilst not ideal, the other options are 1) adding a flat segment outside of the boundary, which leads to a slight sigmoidal shape at the boundary after convolution and 2) continuing the curve outside of the boundary, which is physically unrealistic.

### Estimating beam size

Where circular/elliptical or square/rectangular beams are used, the beam size is either known, or can be directly measured from photomicrographs. Otherwise, the beam size can be determined using a profile measured over a known step function, either real or simulated.

The diameter of a circular beam can be obtained from a profile made over a known step function by approximately running the methodology presented in Fig 3 in reverse. The width of a square or rectangular beam is determined by calculating a moving mean from a step function, then calculating the window size necessary for fitting the moving mean to the data representing a convoluted known step function, which should be three linear segments.

Likewise, where the beam is Gaussian, Lorentzian or Voigt, the size can be estimated or directly measured. For a Gaussian beam, a measured profile over a step function is fitted to an equation with the form:

$$\text{C}(\text{x})={\text{C}}_{2}+({\text{C}}_{1}{-\text{C}}_{2})\times \frac{1}{2}(1+\text{erf}(\frac{\text{x-X}}{\sqrt{2}\sigma }))$$ (7)

and for a Lorentzian beam:

$$\text{C}(\text{x})={\text{C}}_{2}+({\text{C}}_{1}-{\text{C}}_{2})\times (\frac{1}{\pi }\text{arctan}(\frac{\text{x-X}}{\gamma })+\frac{1}{2})$$ (8)

C_1_, C_2_, x and X have the same meanings as in eq (2). For the Gaussian (G), the full width at half maximum (FWHM, Γ) is 2√(2ln2)σ, and Γ = 2ɣ for the Lorentzian (L). For beams best approximated by Voigt shapes, pseudo-Voigt (pV) profiles are simulated from a linear combination of (7) and (8):

$$\text{C}({\text{x},\Gamma )}_{\left[\text{pV}\right]}=\eta \text{C}({\text{x},\Gamma )}_{\left[\text{G}\right]}+(1-\eta )\text{C}({\text{x},\Gamma )}_{\left[\text{L}\right]}$$ (9)

[18]. FWHM (Γ) of the pseudo-Voigt (Γ_[pV]_) line is determined from those of the Gaussian (Γ_[G]_) and Lorentzian (Γ_[L]_) lines:

$$\begin{array}{l} \Gamma_{\left[pV\right]}=(\Gamma_{\left[G\right]}^{5}+2.69269\Gamma_{\left[G\right]}^{4}\Gamma_{\left[L\right]}^{\;}+2.42843\Gamma_{\left[G\right]}^{3}\Gamma_{\left[L\right]}^{2}+4.47163\Gamma_{\left[G\right]}^{2}\Gamma_{\left[L\right]}^{3}\\ +0.07842\Gamma_{\left[G\right]}^{\;}\Gamma_{\left[L\right]}^{4}+\Gamma_{\left[L\right]}^{5}{)}^{\frac{1}{5}} \end{array}$$ (10)

and η is estimated as:

$$\eta =1.36603(\frac{\Gamma_{\left[G\right]}}{\Gamma_{\left[\text{pV}\right]}})-0.47719{\left(\frac{\Gamma_{\left[L\right]}}{\Gamma_{\left[\text{pV}\right]}}\right)}^{2}+0.11116{\left(\frac{\Gamma_{\left[L\right]}}{\Gamma_{\left[\text{pV}\right]}}\right)}^{3}{}^{}$$ (11)

[19]. All of the above methods are incorporated into PACE-GD.

**Fig 4:**
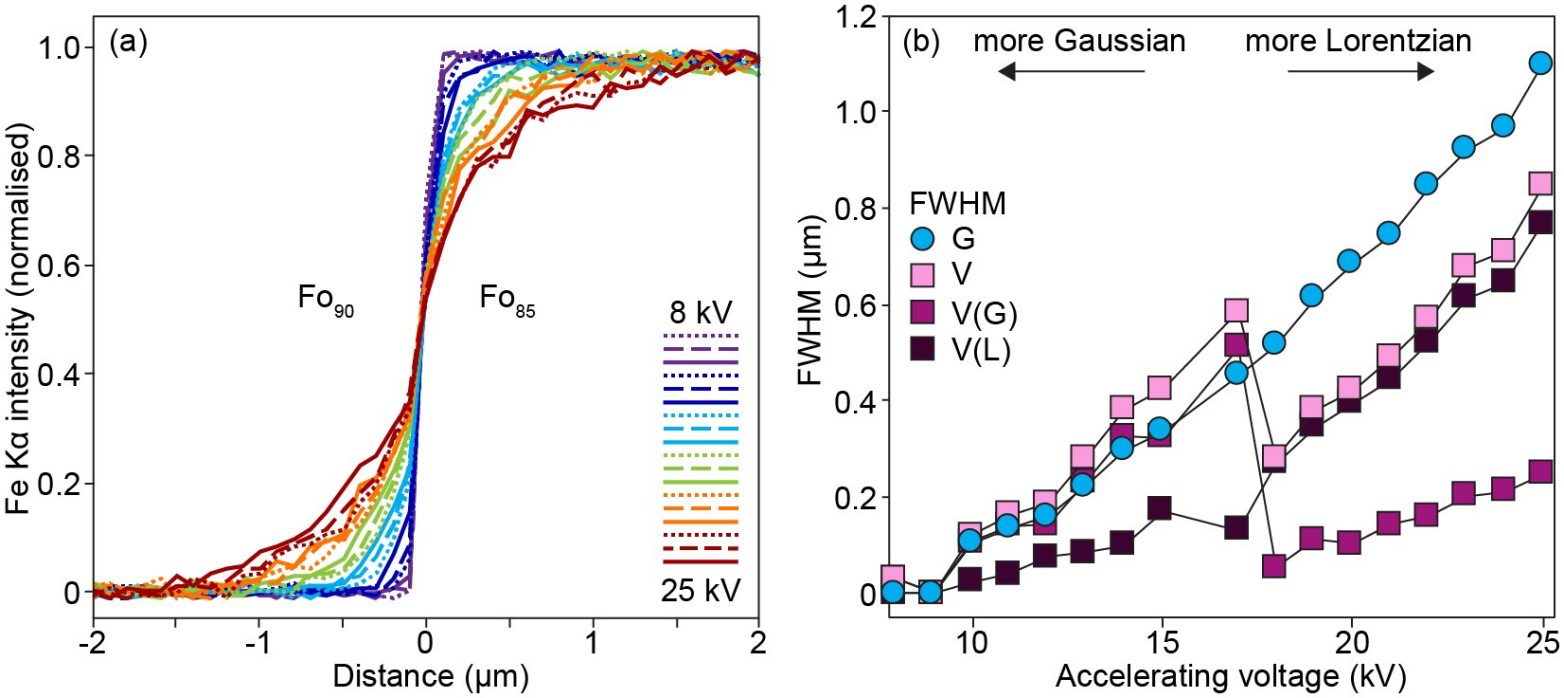
The effect of EPMA accelerating voltage on a step function measured over a Fo_85_-Fo_90_ couple, simulated using the Monte Carlo method implemented in CASINO (20). (a) profiles of Fe Kα (counts, normalised) as a function of accelerating voltage, showing an increase in width, but also a change in shape, as the kV increases. (b) the profiles from (a), deconvoluted using PACE-GD to give the FWHM as a function of kV. This was done both assuming a purely Gaussian interaction (circles) as well as a pseudo-Voigt interaction (squares). The FWHM of the pseudo-Voigt line is shown (V), as well as those of the Gaussian (V(G)) and Lorentzian (V(L)) components comprising the Voigt line. Lines (dashed, dotted and solid) represent increasing intervals of 1 kV.

Where a profile over a couple with a perfect step function, either experimental or natural, cannot be directly measured, software packages allow the direct simulation of electron interactions and thus the extraction of beam size (e.g. CASINO [20]). For example, consider a case where Fe-Mg profiles are measured by electron microprobe (e.g. [21–24]. A couple between Fo_85_ and Fo_90_ (i.e. Mg/(Mg+Fe) = 0.85 and 0.90, respectively) was simulated using CASINO v2 at a series of accelerating voltages, then the resulting profiles fitted using PACE-GD, both assuming a Gaussian beam and assuming a pseudo-Voigt shape. According to the simulation, the assumption of a Gaussian interaction is valid up to ~15 kV, after which the interaction develops wider tails and becomes more Lorentzian in shape, reflected in the relative FWHMs of the Lorentzian and Gaussian components of the pseudo-Voigt interaction volumes (Fig 4). The advantage of the empirical deconvolution method shown in Fig 3 is that any such interaction type can be simulated, without the requirement that the interaction volume is strictly Gaussian, as required when using eq (1).

Aside from convolution effects associated with interaction volumes, there are also effects associated with secondary fluorescence in EPMA. This is associated with measuring low concentrations of elements in phases adjacent to a phase with a much higher concentration of the same element [25, 26], such as Ti in quartz adjacent to rutile [27]. The method in PACE does not account for this phenomenon. The extent of this effect will be sample specific, and specific to the analytical conditions used.

## Example applications

These tools have applicability both where experimental diffusion profiles are measured (e.g. in experimental petrology, materials science) and for natural diffusion profiles (often measured in petrology, volcanology, etc). Some example applications are presented below.

### Experimental Ti diffusion profiles in quartz

Ti diffusion in quartz has had wide ranging applications, including determining timescales of 1) magma chamber processes [3, 28–30]; 2) metamorphic cycles [31] and 3) porphyry formation [32], as well as for proposing a relatively low granite solidus [33]. Therefore, the accurate determination of Ti diffusivities in quartz is of considerable importance.

**Fig 5:**
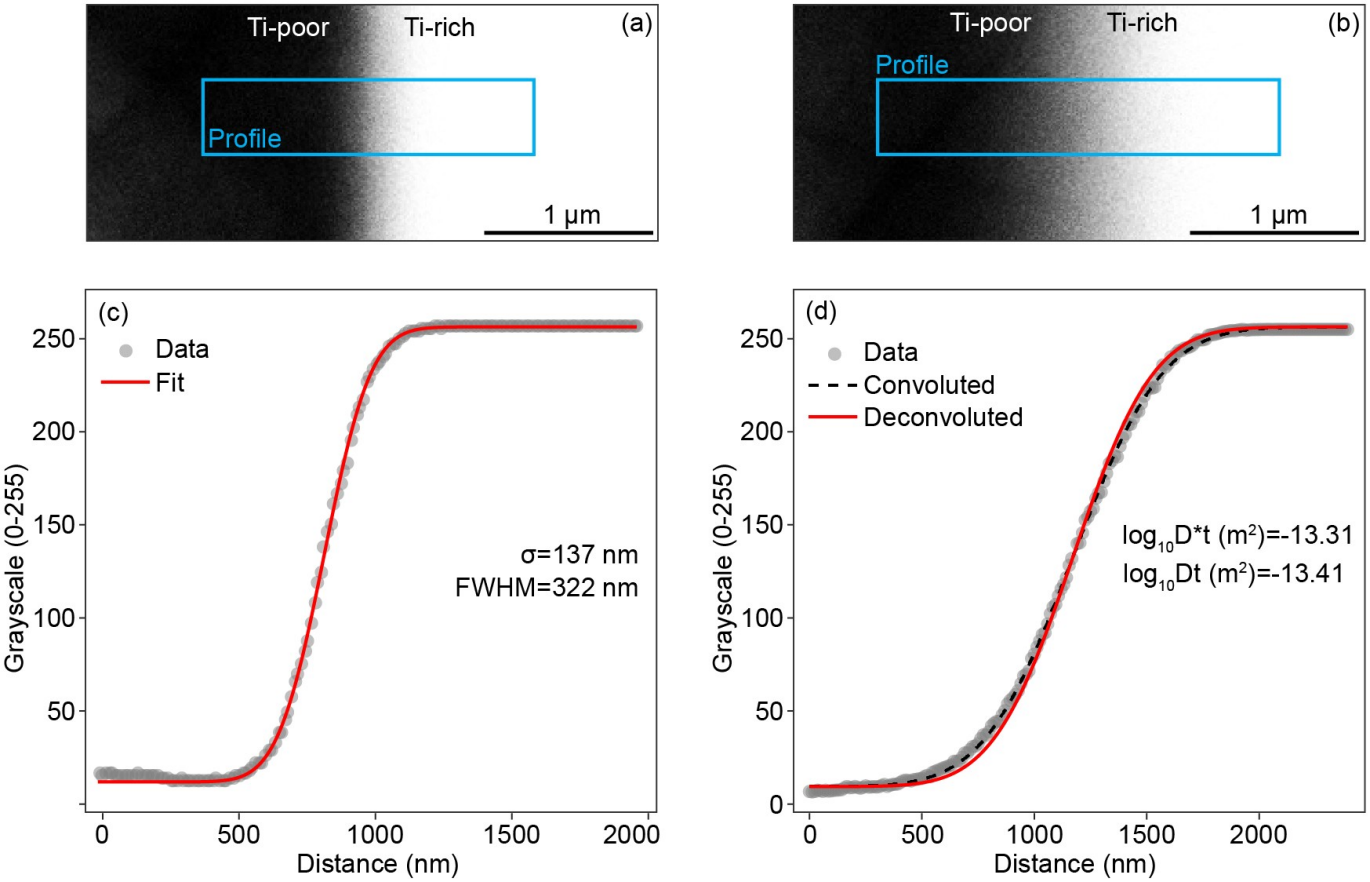
Deconvoluting Ti profiles in quartz determined by SEM-CL. Images were acquired using a Zeiss Gemini 1530 field emission gun scanning electron microscope equipped with an ellipsoidal mirror and an ASK SEM-CL View VIS (250–900 nm) imaging spectrometer. The SEM was operated at 7 kV, 10 nA with a working distance of 14.2 mm. (a) CL image of an un-annealed high Ti quartz-low Ti quartz couple, with an extracted greyscale profile in (c). (b), same sample as in (a), after annealing at 1600°C, 20 kbar for 89.5 h, showing a wider transition zone, with the extracted profile in (d). (c) assuming that the gradient in (a) is purely convolution (i.e. (a) is a step function) and the beam-sample interaction is Gaussian, the FWHM is determined by fitting a curve with the form erf(x/(√2σ)) to the data, giving FWHM = 322 nm. (d) extracted profile from (b), deconvoluted using PACE and the FWHM from (c). The ‘Ti-rich’ section contains ~3000 wt. ppm Ti, versus <0.2 wt ppm for the ‘Ti-poor’ section.

Fig 5 shows cathodoluminescence images of two quartz pieces, both including a low Ti core (<0.2 wt. ppm) over which a high Ti rim (~3000 wt. ppm) was grown experimentally (both images courtesy of Andreas Audétat at the Bayerisches Geoinstitut, Germany). The couple in Fig 5a shows the sample directly after synthesis (1000°C, 1 kbar, 5 hours). The couple in Fig 5b shows the same sample after annealing at 1600°C, 20 kbar, for 89.5 h, to induce diffusive Ti flux. Fitting eq (7) to five grayscale profiles extracted (using ImageJ [34]) from the interface between the high and low Ti sections of the non-annealed couple (Fig 5a) gives a mean σ = 137 nm, hence FWHM = 322 nm (determined by PACE-GD). Applying the same FWHM to a profile extracted from the annealed couple gives log_10_Dt (m^2^) = -13.41(±0.01) (deconvoluted), with the original fit giving log_10_D*t (m^2^) = -13.31(±0.01). As t = 89.5 h, log_10_t (s) = 5.51 so log_10_D (m^2^s^-1^) = -18.92. Interestingly, this diffusion coefficient is lower than extrapolations of both previous experimental determinations of Ti diffusion in quartz [35, 36]. This could be an effect of pressure, composition or diffusion mechanism, but this does not affect the validity of the deconvolution routine.

### Magmatic timescales from the Bishop Tuff

Volcanic quartz crystals often preserve zoning in Ti content, interpreted to be formed by changes in titania activity or temperature, or be the consequence of disequilibrium during growth [37–41]. In any case, the Ti zoning leads to intra-crystalline chemical potential gradients, which drive diffusive flux. The spatial extent of the diffusion profiles can then be used to understand the temperature-time history of the magmatic system. Gualda et al. [42] presented 20–30 μm long profiles measured using synchrotron X-ray microfluorescence with a stated 5 μm beam size, assumed to represent the FWHM of a Gaussian beam-sample interaction (e.g. [43]). Applying PACE to their data shows a minor convolution effect, resulting in a Dt shift of 0.06 log units, which equates to a time decrease of just ~13%. This effect can be considered negligible given reasonable uncertainties associated with diffusion modelling, which, for this system, mainly include 1) temperature; 2) whether samples are prepared with a plane exactly perpendicular to the compositional boundary; 3) discrepancies between experimental determinations of diffusion coefficients [35, 36] and 4) initial conditions.

**Fig 6:**
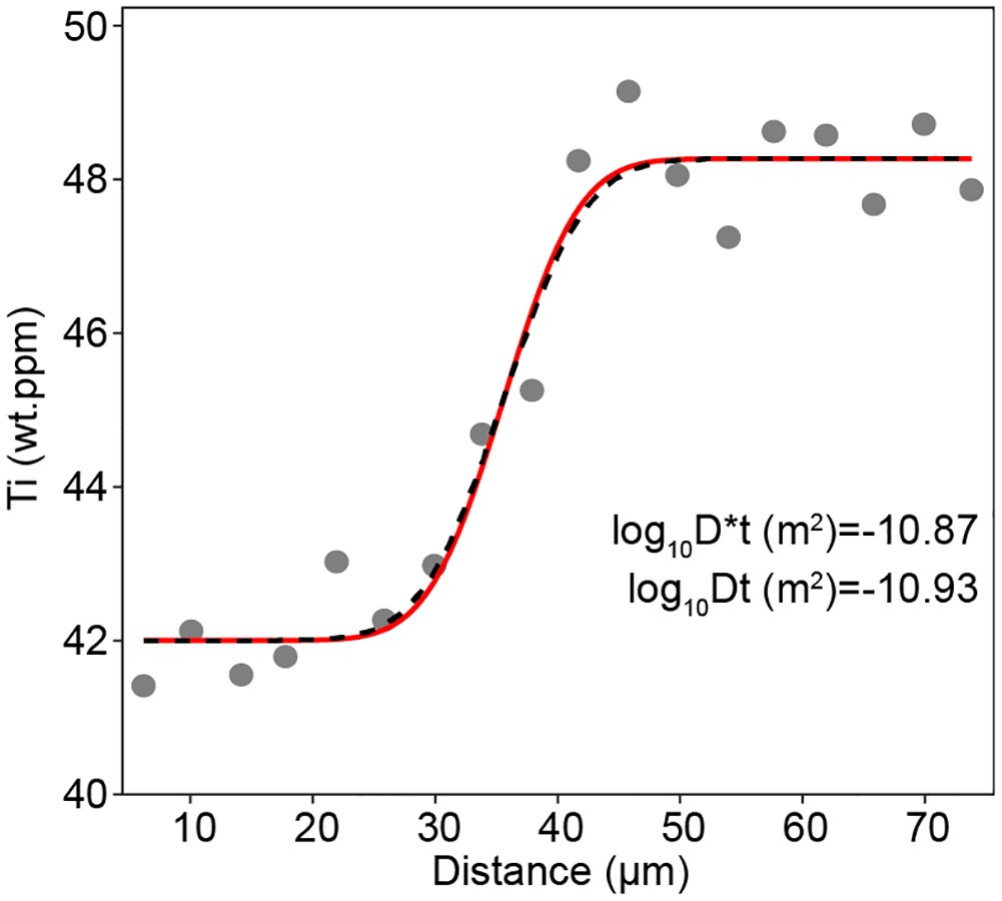
Data from Gualda et al [42], fitted and deconvoluted using PACE, assuming a 5 μm FWHM Gaussian beam. The measured Dt (D*t) is just 0.06 log units greater than the true Dt (Dt), i.e. convolution has almost no effect on the measured profile.

In this case, given the profile and beam types, the result can be compared to the exact formulation in eq (1), which gives log_10_D*t (m^2^) = -10.86 (c.f. -10.87 in Fig 6). The former is exact, whereas the latter is a numerical approximation, hence the small discrepancy.

### Experimental diffusion profiles measured using a rectangular laser beam

**Fig 7:**
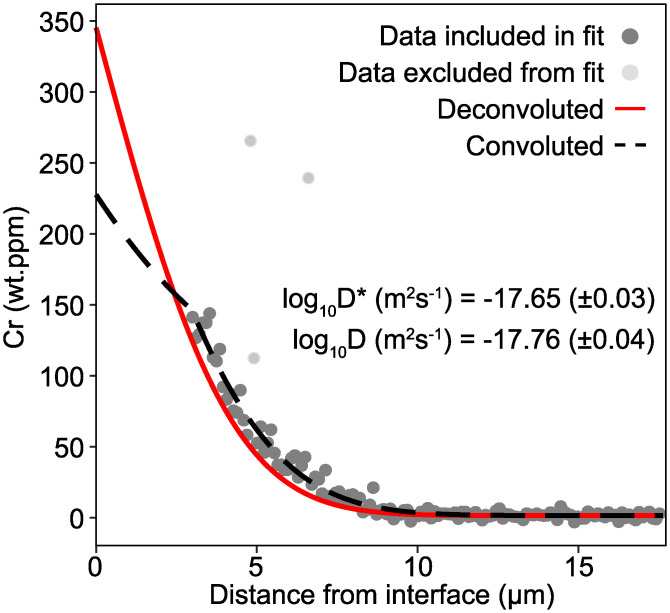
An experimental Cr diffusion profile in forsterite, measured using LA-ICP-MS with a 6x100 μm, deconvoluted. The measured D (D*) is 0.11 log units higher than the deconvoluted D. Note the deviation between data and model in the near-interface region—this is due to the way that PACE treats boundaries (fixed composition equal to the rim composition).

Slit-shaped beams are now routinely used in laser ablation inductively-coupled plasma mass spectrometry (LA-ICP-MS) to measure concentration profiles in minerals and glasses [44–47]. In this technique, a slit-shaped aperture is placed in the optical pathway and rotated to have its long axis parallel to the diffusion interface. The sample is then moved beneath the stationary beam, continuously passing data to a mass spectrometer, creating a profile of concentration versus distance. Jollands et al. [47] presented an experimental data set of Cr diffusion in olivine measured in this manner. The diffusivity of Cr was shown to be highly anisotropic in olivine, with considerably higher diffusivity parallel to [1] than [100], and also dependent on the externally-buffered silica activity, with higher silica activity giving higher diffusivities. Together, profiles measured parallel to [100] following low silica activity experiments were often short, which in this case means <10 μm. One such profile is shown in Fig 7, with a ~9 μm long Cr diffusion profile, measured using a 6 μm wide (by 100 μm long) laser beam. This was following an experiment at 1306°C, for ~35 days. The convoluted log_10_D* (m^2^s^-1^) = -17.65 ± 0.03. Following deconvolution (Fig 7), log_10_D (m^2^s^-1^) = -17.76 ± 0.04, resolvably different from the convoluted data. The advantage with this method is that, because the length scale of a diffusion profile is broadly proportional to the square root of time, if such short profiles can be deconvoluted, then this opens up the possibility for running shorter experiments and/or experiments at relatively low temperatures.

### Experimental Pb in zircon profiles measured by EPMA

An accurate determination of the diffusivity of Pb in zircon is essential for determining the closure temperature associated with U-Pb dating. Cherniak and Watson [48] presented a profile of Pb-out diffusion from zircon measured by EPMA to supplement a large dataset of Pb diffusivities measured by Rutherford Backscattering Spectroscopy. The measured profile was ~6 μm long, measured with a 25 kV beam. A CASINO simulation of a line profile measured at 25 kV, over a high Pb—low Pb zircon couple gives an error function form (i.e. a Gaussian beam) with σ = 693 nm, i.e. FWHM = 1633 nm. However, because Cherniak and Watson [48] measured on a section 30° from obliquity with the diffusion interface, the interaction volume is compressed by approximately a factor of two in the direction of diffusion. Thus, a FWHM of 816 nm was used for deconvolution.

**Fig 8:**
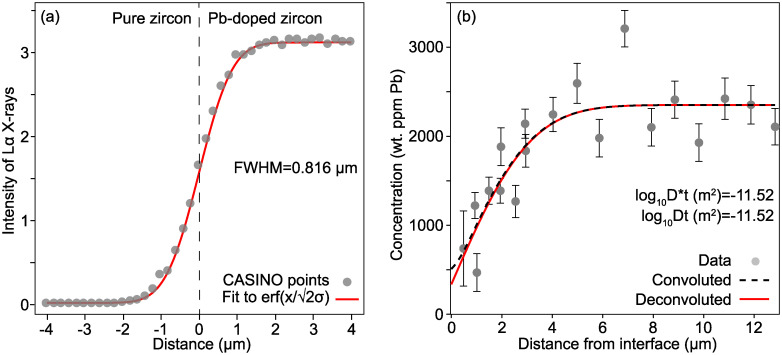
Deconvoluting a Pb in zircon profile from [48]. (a) shows an apparent Pb Lα profile between Pb-doped and Pb-free zircon, generated by CASINO [20], using an incident beam at 25 kV with a nominal 2 μm diameter. From the fit, σ = 693 nm, hence FWHM = 1633 nm. However, because [48] analysed a section 30° from normal to the interface, the relevant FWHM is 1633/~2 ≈ 816 nm. (b) shows the [48] EPMA profile, deconvoluted. Convolution has an effect of <0.01 log_10_D units in this case.

Deconvoluting the measured profile (Fig 8) shows that the effect of artificial profile lengthening is only 0.01 log_10_Dt units in this case, far smaller than the uncertainty from curve fitting. That this effect is so small, even when using a 25 kV beam, is due to a combination of the high stopping power of zircon and the measurements having been done on a non-oblique section.

## Concluding remarks

The PACE software packages allow rapid, simple determinations of analytical convolution artefacts associated with measuring diffusion profiles, either experimental or natural. Whilst the majority of experimental and natural profiles are comfortably much longer than the beam size used to measure them, profiles approaching analytical spatial resolution are becoming more common in the literature. PACE simplifies the extraction of quantitative data from such profiles, and also enables a rapid *diffusion profile* versus *no measurable diffusion profile* distinction to be made. Such tools will become more important as diffusion modelling is used to determine increasingly short timescales.

## Supporting information

S1 File (ZIP)

S2 File (ZIP)

S3 File (PDF)

S4 File (ZIP)
