# Supplementary figures and images for: Assessing analytical convolution effects in diffusion studies: Applications to experimental and natural diffusion profiles
Source: PLoS One. 2020 Nov 24;15(11):e0241788. doi: 10.1371/journal.pone.0241788 (PMC7685509; doi:10.1371/journal.pone.0241788)

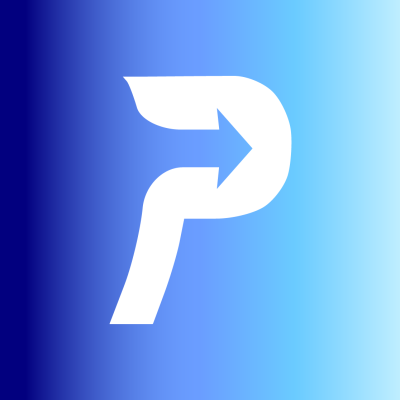

Supplement: S1 File — (ZIP) [file pone.0241788.s001.zip › PACE_installer_MACOSX.app/Contents/Resources/splash.png]
