# Supplementary material for: Assessing analytical convolution effects in diffusion studies: Applications to experimental and natural diffusion profiles
Source: PLoS One. 2020 Nov 24;15(11):e0241788. doi: 10.1371/journal.pone.0241788 (PMC7685509; doi:10.1371/journal.pone.0241788)
Supplement: S3 File — (PDF) [file pone.0241788.s003.pdf]

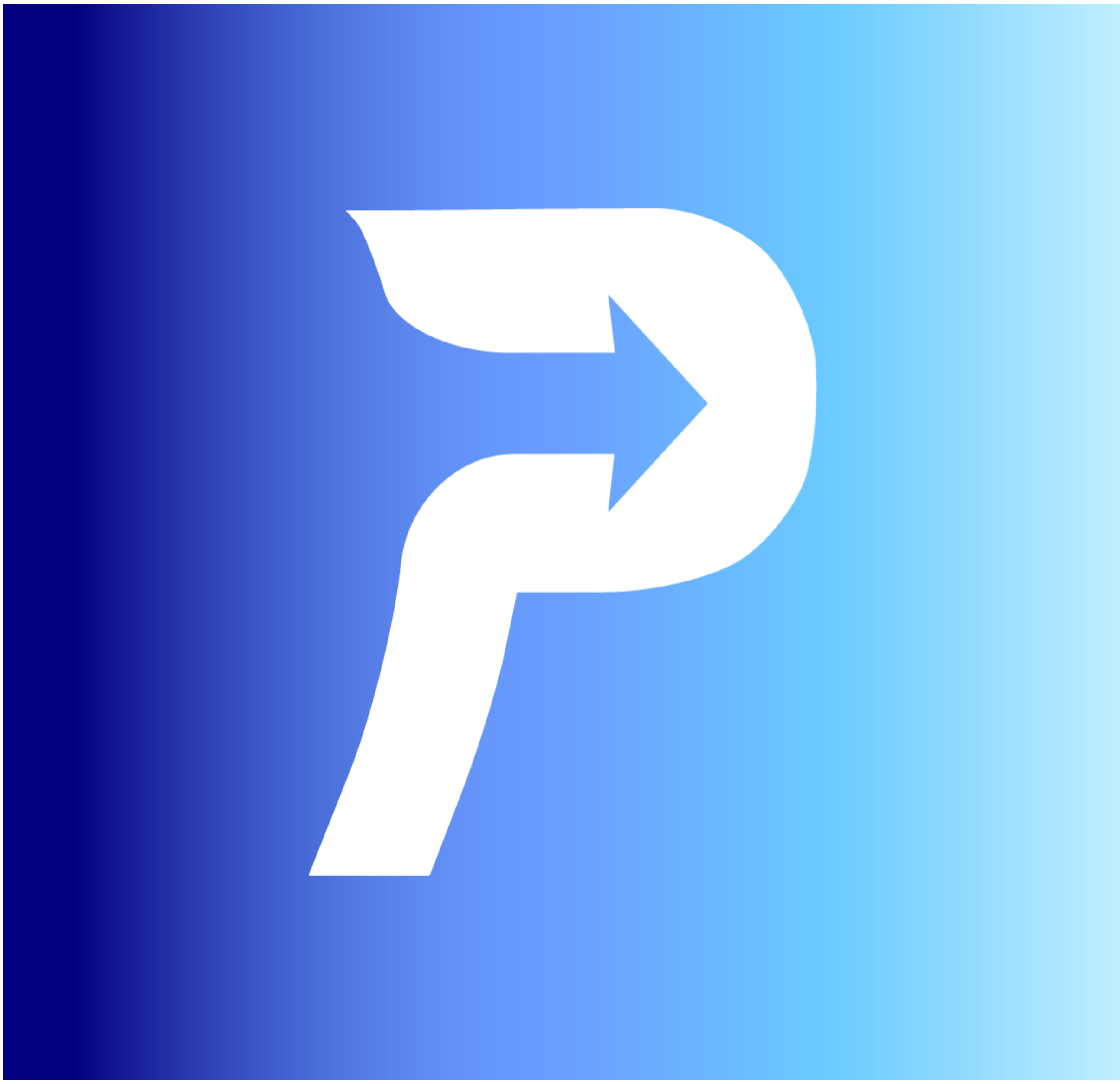

PACE (**P**rogram for **A**ssessing **C**onvolution **E**ffects in diffusion studies)

Manual

for PACE 1.6
PACE-IC 1.2
PACE-GD 1.2

Mike Jollands

1 About PACE

PACE, PACE-IC and PACE-GD have been created to easily assess the effects of beam convolution in diffusion studies. Because the techniques used to analyse diffusion (or other) profiles always have some finite size (i.e., not zero), there will always be some amount of smearing out of the profile. In most cases, this is negligible, because the beam size should be tailored to the diffusion profile length – we generally would not even attempt to analyse a 10 µm profile with a 15 µm beam, for example. However, in some cases, these effects become important.
The original application was PACE, the other two have been built to extend the functionality range.
PACE is built in MATLAB using the functions provided by GUIDE, and built into a standalone application using the MATLAB compiler. Whilst PACE was designed on mac OSX, it has been compiled both for Windows and mac. If you run PACE on Windows, and find issues (especially involving layout), please let me know.
PACE can be run independently without a MATLAB license, which requires some MATLAB runtime toolboxes to be downloaded. This is recommended for people who do not use MATLAB. PACE can also be run directly from MATLAB. This requires version 2019a or higher, and also requires the Statistics and Machine Learning toolbox along with basic MATLAB functions. A translation of PACE into Python is planned.
Whilst the main function of PACE is assessing convolution effects, it can also be used for basic curve fitting. In addition, PACE can produce figures with considerable flexibility in terms of design. These figures can all be exported directly in either vector or raster format.
Whilst care has been taken to ensure PACE works properly using various platforms/screen resolutions/processors, there are likely to be some bugs. If these cannot be fixed by closing and re-opening the app, please get in touch via www.mikejollands.com

PLEASE NOTE: This manual contains repetition, much of the text in the main 'PACE' section has been copy-pasted into the 'PACE-IC' section, and only slightly modified.

1 Getting started with PACE

1.1 Downloading and Installation

1.1.1 Through MATLAB

The recommended way to use PACE is directly through MATLAB. However, for this, you will need at least MATLAB 2019a, including the statistics and machine learning toolbox. PACE will start on older versions of MATLAB, but import/export functionality will not work. The solvers incorporated into the statistics and machine learning toolbox are essential for the curve fitting used in PACE – it will not function without them.
To run on MATLAB:
1) Download the zip file PACE_matlab.zip
2) Unzip – this will give eight files.
3) Place these files into your MATLAB folder, or whatever is your current directory. The current directory can be found by typing **cd** into the command line.
4) In the MATLAB command line, type PACE_mainscreen.m

1.1.2 Mac install

1) Download the zip file PACE_Installer_mac.zip
2) Unzip the contents
3) Follow the installation guidelines, which will require MATLAB runtime to be downloaded, if you don't already have it.
4) If you install into your applications folder, the software will be available under Applications/PACE/application/PACE

1.1.3 Windows install

1) Download the file PACE_Installer_Windows.exe.zip
2) Unzip the contents
3) Run the installation – this will require MATLAB runtime to be downloaded and installed, if you do not already have it.
4) PACE should then be directly available from your start menu.

1.2 PACE Start Screen

The first window to open (after the splash screen) is the PACE start screen, from which other PACE apps can be opened. At this point, the options are PACE, PACE-IC and PACE-GD. Clicking on any of these icons will open up a new window – the start screen remains open.

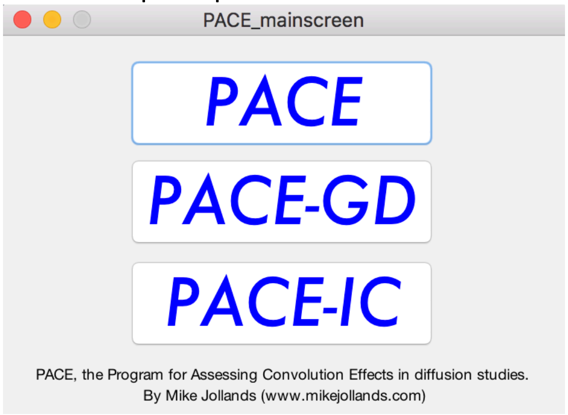

2 PACE

2.1 Main screen layout and general workflow

PACE works from top to bottom and left to right. At the start, only the first two buttons are available (Import and Random), then as the user moves through the program, options become progressively available below, and previous options become unavailable. It is always possible to go back to the previous step using the yellow reset buttons. The two graphs on the right-hand side will be populated during the processes.
Brief help descriptions are available by hovering the mouse over each button. All figure editing options, as well as export options, become available by pressing the cog wheel button in the lower right corner.

2.2 Brief description of components

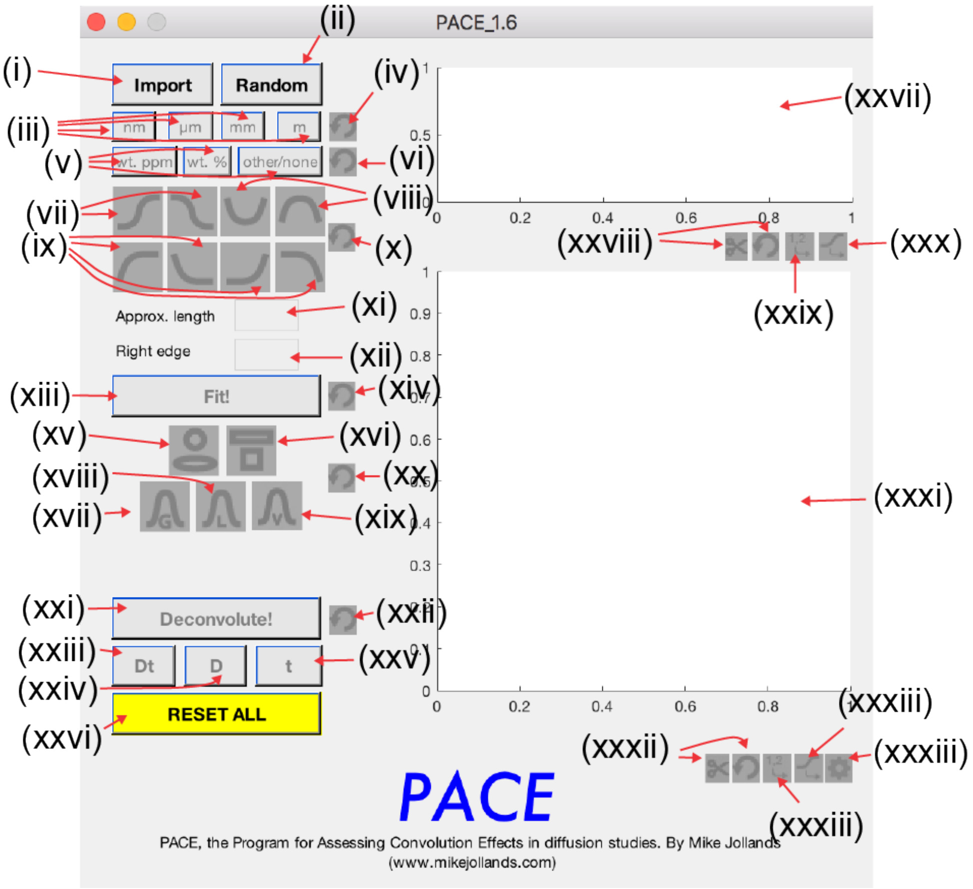

(i) Import button (Section 2.3, p. 6)
(ii) Random data (Section 2.3, p. 6)
(iii) Select the distance units (Section 2.4, p.7)
(iv) Reset the distance units (Section 2.4, p.7)
(v) Select the concentration units (Section 2.5, p. 8)
(vi) Reset the concentration units (Section 2.5, p. 8)
(vii) Sigmoidal curves (Section 2.6.1, p. 9)
(viii) U or upside-down-U shapes (Section 2.6.3, p. 10)
(ix) Half error function shapes (Section 2.6.2, p. 10)
(x) Reset shape choice
(xi) Add the approximate profile length (Section 2.7.1, p. 11)
(xii) Add the position of the right edge (if necessary/if desired) (Section 2.7.2, p. 11)
(xiii) Fit the data to the model (Section 2.8, p. 12)
(xiv) Reset the fit
(xv) Select a circular or elliptical beam shape (Section 2.11.2, p. 15)
(xvi) Select a square or rectangular beam (Section 2.11.1, p. 15)
(xvii) Select a Gaussian beam shape (Section 2.11.3, p. 15)
(xviii) Select a Lorentzian beam shape (Section 2.11.4, p. 15)
(xix) Select a Voigt (pseudo Voigt) shape (Section 2.11.5, p. 15)
(xx) Reset the curve shape
(xxi) Deconvolute the data (Section 2.12, p. 16)
(xxii) Reset the deconvolution
(xxiii) Output base 10 logarithm of the diffusion coefficient multiplied by time ($\log_{10}Dt$) – the default option. (Section 2.14, p. 18)

(xxiv) Output base 10 logarithm of the diffusion coefficient ($log_{10}D$) (Section 2.14, p. 18)
(xxv) Output base 10 logarithm of time ($log_{10}t$) (Section 2.14, p. 18)
(xxvi) Reset all – return to the start
(xxvii) Top axes, used for plotting the first fit.
(xxviii) Exclude data, and reset exclusions. (Section 2.9, p. 12)
(xxix) Simple export of fit parameters from first fit. (Section 2.10, p. 13)
(xxx) Simple export of top plot as vector graphic (Section 2.10, p. 13)
(xxxi) Second axis, for plotting deconvolution
(xxxii) Exclude data and reset exclusions from second plot
(xxxiii) Export fit data from lower plot (Section 2.13, p. 17)
(xxxiv) Export lower plot as vector graphic (Section 2.13, p. 17)
(xxxv) Toggle figure modification settings (Section 2.15, p. 19)

2.3 Import / Random data

Initially, you have the choice of either importing, or randomly creating data. The former is more useful for practical purposes, but the latter is designed for testing or for education purposes.

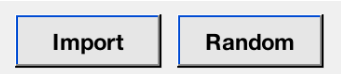

Good to know: 'Import' and 'Random' remain enabled continuously, regardless of what other buttons are pressed. Clicking either will reset PACE.

Pressing random will generate diffusion profiles in the top right graph window. These are generated by creating a diffusion profile with random lengths and concentrations (using an analytical solution to Fick's second law), then applying a random amount of random scatter, thus no two random profiles will ever be the same, e.g.:

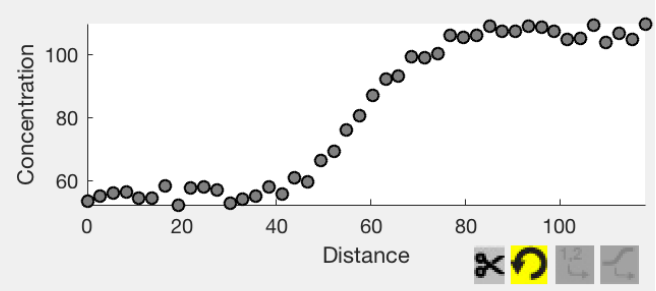

Pressing import will open a standard import window, where any .csv, .xlsx, .xls or .txt file can be imported. Ideally, these should be files with only two columns, where the first column is distance (either from the interface, or along a profile) and the second is concentration. If this is not the case, a second dialog box will appear asking for extra input.

Warning: the Distance and Concentration columns must contain the same number of data points.

Warning: in some complex Excel spreadsheets, there may be cells that appear empty, but are not (e.g. include a space). If such a cell exists somewhere in the same column as the data, PACE will fail to import. This can be solved by copying and pasting the data to be imported into different columns, or, even better, into a new spreadsheet.

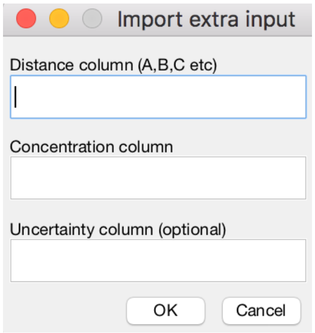

Use Excel-type notation for the columns, e.g. 1st column is A, 2nd is B, 27th is AA, etc:

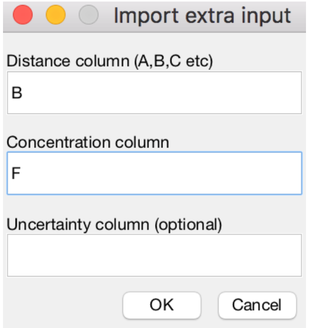

Pressing 'OK' will then import the data, 'Cancel' will return to the main screen. Importing, or randomly generating data, will enable the next line of buttons.

Warning: PACE may struggle if the units of distance are already in metres, for a micrometre/nanometre-long profile, i.e. if the values are very small. It is better to keep the distance units in micrometres, or whatever units are necessary so the profile length is on the order of 1-100, rather than converting to metres before import.

In addition, uncertainties can be added, if desired, using the third box:

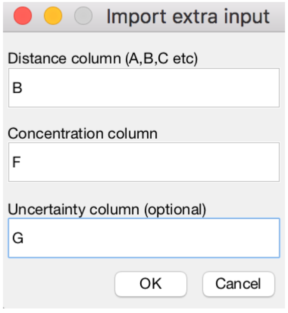

If uncertainties (e.g. σ) are added, they are converted to weights in PACE using $1/\sigma^2$. If weights are added, error bars will be added to the top right scatter plot.

2.4 Select Distance Units

After either importing or randomly generating data, the next line of buttons become enabled, asking for the relevant distance units. The available units are nanometres (nm), micrometres (µm), millimetres (mm) and metres (m), assuming that most geologically-relevant measurements will be in either nm or µm.

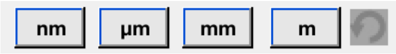

Selecting one of these will cause the others to become disabled and illuminate the reset button on the right:

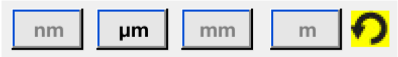

The yellow reset button:

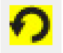

resets the distance units and the graph x-axis.
Additionally, the selection will update the x-axis of the top-right graph:

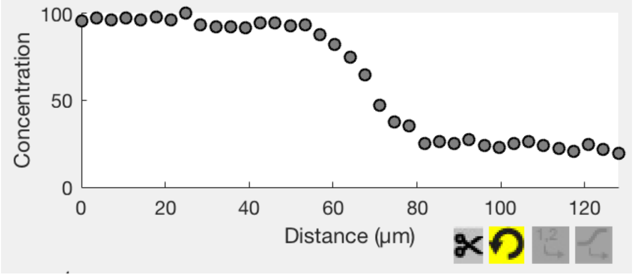

and some other text through the program.
Good to know: The yellow reset button always goes back one step, whereas the 'Reset all' button goes right back to the start. Only one reset button is available at any time.
Selecting a distance unit will enable the next row of options (concentration units)

2.5 Select Concentration Units

After selecting distance units, the buttons allowing concentration units to be selected becomes enabled:

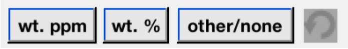

Select one unit. The assumption is that most relevant data will be in wt. ppm. Upon selecting the unit, the graph will update its y-axis:

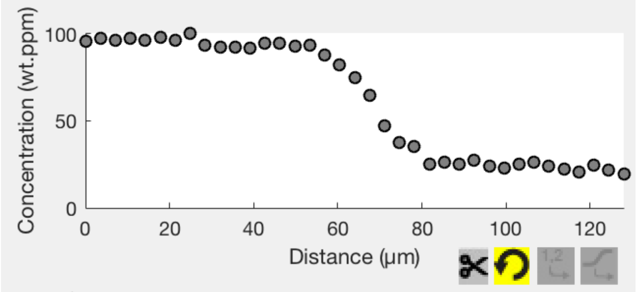

the other options will become disabled and the relevant reset button becomes enabled:

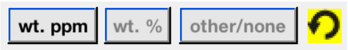

Good to know: given the solutions of the diffusion equation used in PACE, this step *doesn't matter*. This is because PACE assumes diffusion is concentration independent. The step is added simply to have a unit on the y-axis, and because there is a plan to include concentration-dependence in a future version of PACE.

2.6 Select profile type

The next buttons to become enabled allow the solution to the diffusion equation to be selected graphically.

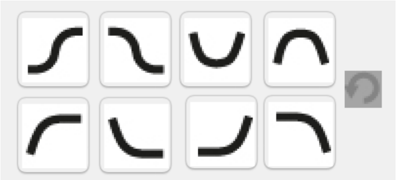

It is recommended that users familiarise themselves with the physical meanings behind the different solutions; brief descriptions are given below. Thorough descriptions are given in Crank (1975) and Carslaw and Jaeger (1959).

Upon selecting a profile type, the other icons will become disabled.

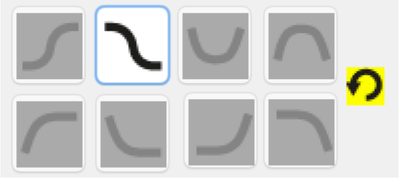

As before, this choice can be reversed using the reset button, which illuminates once a choice is made.

2.6.1 Type 1: sigmoidal profile

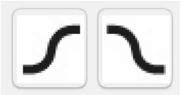

The two top-left shapes represent solutions to the diffusion equation where two semi-infinite 1-D objects, with different concentrations, are placed end-to-end.

Good to know: A *semi-infinite* object/line is limited on one side, and stretches to infinity on the other. Is anything geologically-relevant semi-infinite? No. But this basically means that the size of the object (e.g. the crystal) has to be considerably longer than the length of the diffusion profile.

This assumes that the initial condition is a perfect step function, and diffusion is concentration-independent. If these assumptions hold true, the position of the inflexion point is always at the position of the initial step. Geologically-relevant situations where such a solution is used include those where a crystal has been overgrown by the same type of crystal with the same/similar major element contents, but different trace/minor element contents.

Good to know: Concentration-independent diffusion is generally assumed, but there are numerous examples where it is not valid. These include 1) many inter-diffusion situations (e.g. Fe-Mg in olivine (Chakraborty, 1997; Dohmen et al., 2007; Dohmen and Chakraborty, 2007), Fe-Mg-Mn-Ca in garnet (Chakraborty and Ganguly, 1992; Elphick et al., 1985; Ganguly, 2010; Ganguly et al., 1998)); 2) where the ion brings defects as it diffuses (e.g. trivalent cations in periclase, olivine (Crispin and Van Orman, 2010; Jollands et al., 2018; Van Orman et al., 2009)); 3) where there are two or more substitution mechanisms, with exchange between defects (e.g. Li in olivine (Dohmen et al., 2010)).

The solution, as used in PACE, is:

$$C(x,t) = C_2 + (C_1 - C_2)\times\tfrac{1}{2}erfc\left(\frac{x - X}{2\sqrt{Dt}}\right)$$

where C is concentration, x is distance, t is time, C_1 and C_2 are concentrations on either side of the sigmoid, X is the midpoint (inflexion point) of the sigmoid and D is the diffusion

coefficient. D is conventionally given in m^2s^{-1} , thus the units of x are metres, and t is given in seconds.
Good to know: simulate a normal distribution, add every value to the sum of all previous values, and a sigmoidal curve forms. This curve has the same form as the error function (with different magnitude). This is an important concept, because the normal distribution and diffusion both relate to probability.

2.6.2 Type 2: Half-sigmoid

This solution is similar to the previous, with the form of half a sigmoid.

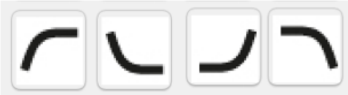

The two leftmost icons are recommended, because these both assume that the interface is at x=0, whereas the rightmost icons assume that the interface is at some arbitrary position x≠0. It is recommended that if the data are in the form where the interface is at x≠0, the data are recast so that the interface is at x=0. This is not necessary, but is generally protocol.
This solution assumes a semi-infinite medium, bounded on one side by the interface. This interface has a constant composition.
Geologically, this might be useful where a crystal exchanges with a fluid or melt, without any movement of the crystal boundary (no dissolution/re-precipitation). It is commonly applied in experiments, where a boundary can be fixed through experimental design.
Where the interface is at x=0:

$$C(x,t) = C_2 + (C_1 - C_2) \times erfc\left(\frac{x}{2\sqrt{Dt}}\right)$$

In this case, C_1 is the interface concentration, and C_2 is the background concentration. This works whether the core has a higher or lower concentration than the rim.

Good to know: the equations given here can be presented in different forms in different papers/references. The complementary error function (erfc) is one minus the error function (erf), so if the same solution uses erf rather than erfc, the beginning of the right-hand side is C_1-… rather than C_2+…. Moreover, the notation given for the concentrations is not standardised – you may see C_1 and C_2; C_0 and C_1; C_{rim} and C_{core}; C_s and C_∞ etc.

For the right-hand icons, the interface is at x≠0. In this case, the x vector is simply reversed, with the interface position added (here as X):

$$C(x,t) = C_2 + (C_1 - C_2) \times erfc\left(\frac{X - x}{2\sqrt{Dt}}\right)$$

2.6.3 Type 3: U or upside-down U

This solution effectively has a type 2 curve at either end of an object with limited extent.

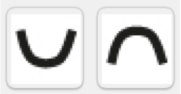

PACE assumes that x=0 represents one of the interfaces, and the other position is either added by the user, or found by fitting.

This solution is described as:

$$C(x,t) = C_2 + (C_1 - C_2)\times\left(erfc\left(\frac{X-x}{2\sqrt{Dt}}\right) + erfc\left(\frac{x}{2\sqrt{Dt}}\right)\right)$$

where X is the position of the second boundary. Note that only one C_1 and one C_2 are given, thus the interfaces have the same concentration by default.
This solution is often used for situations where a relatively small crystal experiences diffusion from two opposite faces.
Warning: this solution only works when the core concentration of the crystal is the same as the initial concentration (within analytical precision). Otherwise, the system needs to be modelled using a different solution, or numerically. Attempts to use this for situations where the background has become modified will give incorrect results, *even if the fits appear reasonable.*

2.7 Extra inputs

Following the selection of a profile shape, one or two text boxes below become editable. In most cases, filling in these boxes is optional – if not, a prompt will appear.

2.7.1 Approximate length box

This box will always be editable, it allows the user to approximately estimate the diffusion length-scale. This may be necessary, but generally is not, given that PACE makes use of a multistart solver. An order-of-magnitude estimate is sufficient. This guides the non-linear least squares regression used below.

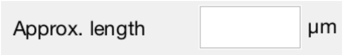

Good to know: the solvers used in PACE have been modified such that they have several start points. This enhances the probability of finding a global minimum.

2.7.2 Right edge position box

This box allows the user to add in the position of the interface at the right-hand edge of the profile, where the interface is not at x=0.
This box becomes available if any of the four right-hand icons are selected. It is optional for the U or upside-down-U shapes, and **mandatory if one of the two lower-right icons is selected**.
For example, consider the following random data:

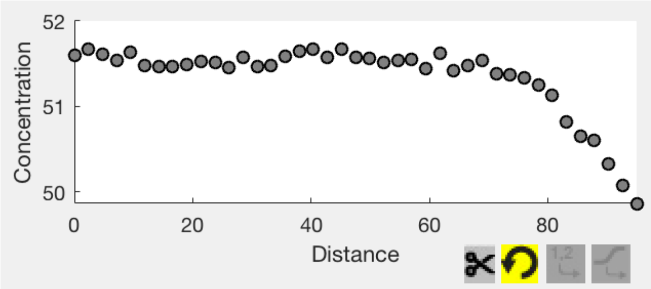

In this case, the maximum distance value of the data is ~95, but let's assume that the system extents 10 µm past the furthest data point. Then, we type 103 into the right edge position box:

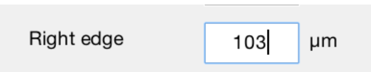

PACE will not allow the user to progress if one of these icons is selected and a right-hand position is not given. In this case, the following will appear:

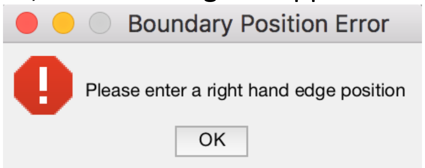

2.8 Fit button

Following the selection of a profile shape, and the (generally) optional addition of extra information, the first fit can be done by pressing the Fit button:

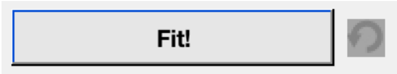

After pressing fit, a curve will appear over the data on the top right graph. The appearance of this cannot be modified. In addition, the value of Dt will be presented as a base 10 logarithm in the plot:

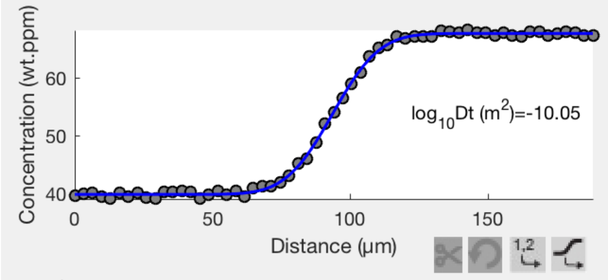

This fitting is done by non-linear least squares regression, using the nlinfit solver in MATLAB. Weighting is added if required. Like all non-linear least squares regression, the starting guess is important for determining the final outcome – the solver may find a local minimum rather than a global minimum. If you find PACE is failing to fit the data, try adding an approximate profile length, as described above:

Good to know: you can change the approximate profile length, then press fit again, without going back. The same goes for changing the position of the right edge.

Pressing the reset button next to the ‘Fit!’ button will remove the fitted curve from the top right plot.

2.9 Exclude and reverse

These two buttons at the lower right corner of the top right graph allow data to be excluded from consideration and allow this exclusion to be reversed.

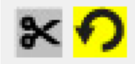

Note that the same icons exist at the bottom of the lower graph too, so if you press the button below the top graph, please do not try to exclude data from the lower graph (and vice versa). The graphs are linked, so any changes made in one will automatically affect the other.

Good to know: exclusion can be done before or after pressing ‘Fit!’. It is also possible to fit, exclude, then fit again, if you suspect one point is leading to erroneous fitting.

Clicking the scissors:

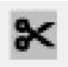

enables point removal. At this point, the cursor will become a cross when hovered over the graph. Clicking and dragging will form a red-bordered rectangle. Points selected inside this rectangle will turn red.

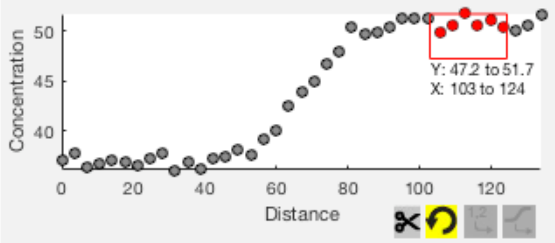

To remove these from consideration, **press the spacebar** (some other keys work as well)**.** At this point, the points become light grey, which means they have been removed from consideration. Press ‘Fit!’ again to refit without the excluded data.

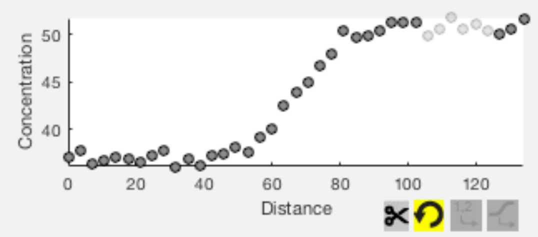

To bring the excluded data points back, press the yellow circular arrow:

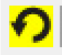

Which returns the points to the original dark grey colour:

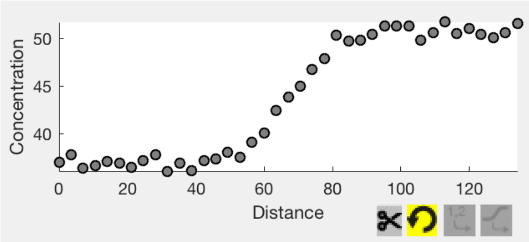

2.10 Export from the top plot

Many options are available for exporting data from the lower plot (discussed below). From the top plot with the first fit, only two options are available,

2.10.1 Export fit data

This copies the $\log_{10}$Dt, C1 and C2 values to the clipboard.
This is done simply, after fitting, by pressing the icon to the right of the two red icons. This icon only becomes available after fitting has been done.

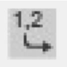

For example, following this fit:

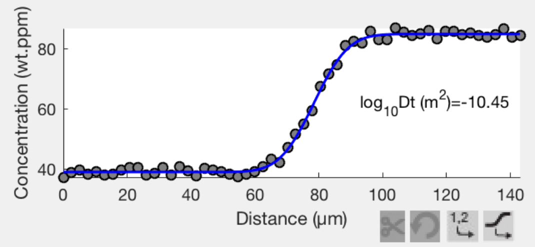

Pressing the export button, then pasting into Microsoft Excel gives:

	A	B
1	-10.449	0.0692562
2	38.9976	0.463715
3	84.8629	0.525875

This makes use of the num2clip function written by Grigor Browning. The value in cell A1 is $\log_{10}Dt$ (m^2), as given in the plot. The values in A2 and A3 are concentrations C1 and C2. The values in B1, B2 and B3 are the ±95% confidence limits. These confidence limits are calculated from the vector of residuals and the Jacobian from the nlinfit function, and determined by MATLAB's nlparci function.
Whilst this export is not the main function of PACE, it may be useful for quickly processing diffusion data where convolution effects are demonstrably negligible.

2.10.2 Export plot

For a quick export of the top plot, use:

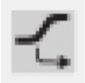

This will export the top plot as a vector graphic to the clipboard. Pressing this button, then pasting into Adobe Illustrator gives:

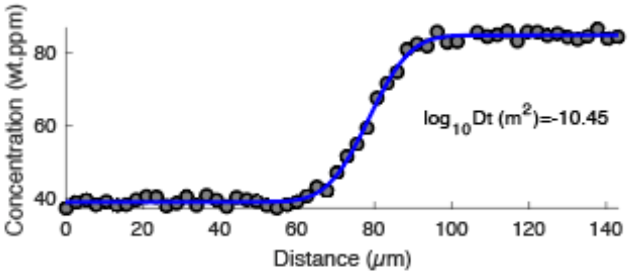

These objects can all then be manipulated as desired.

2.11 Selecting beam type

After fitting, the five icons showing beam shapes become available. The shapes supported by PACE are circular/elliptical, square/rectangular, Gaussian, Lorentzian and Voigt.

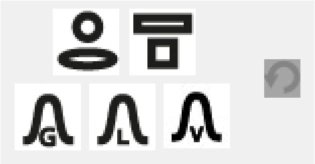

Upon selecting one of the beam types, the others become unavailable and reset becomes illuminated. As before, the choice can then be repeated using the reset button.

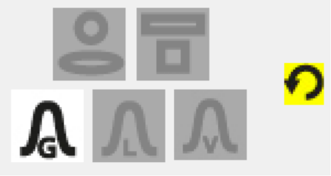

When any button is pressed, a dialog box opens, asking for further input. This is where you tell PACE the beam size. PACE-GD may help to determine this value, where it is not known.

2.11.1 Square/rectangular

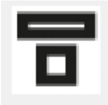

For square/rectangular beams, the width is required:

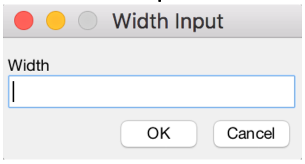

2.11.2 Circular/elliptical

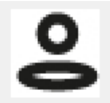

For the circular/elliptical beams, the diameter is needed

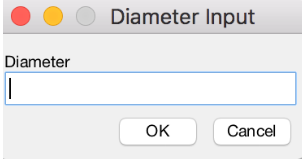

2.11.3 Gaussian

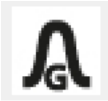

For Gaussian beams, the full width at half maximum (FWHM) is needed:

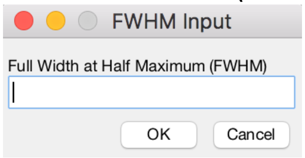

2.11.4 Lorentzian

The requirement is the same as for Gaussian beams (the FWHM)

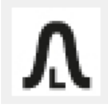

2.11.5 Pseudo-Voigt

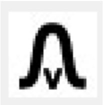

For beams best approximated by pseudo-Voigt shapes, the FWHM of both the Gaussian and Lorentian components is needed:

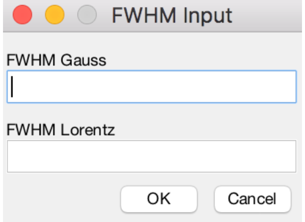

2.11.6 Input reminder

Once the beam size is given, text appears below the beam shape selection icons reminding the user of the inputted value, e.g.:

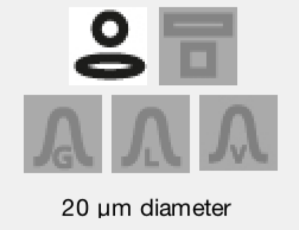

2.12 Deconvolution

The main functionality of PACE is contained in the 'Deconvolute' button. This refits the data using the given beam type and size, and gives an estimate of the true (***deconvoluted***) diffusion profile, along with the best fit to the measured (***convoluted***) profile. The nomenclature basically assumes that all measured profiles are convoluted to some degree, which is either negligible, or important.

Upon pressing deconvolute:

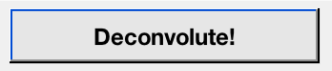

a wait-bar appears:

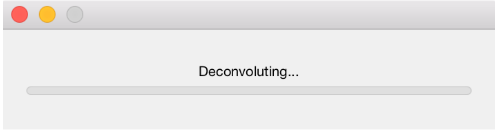

The wait bar normally takes ~1-2 seconds to complete. The delay is because PACE employs a multi-start solver to maximise the chances of obtaining a global minimum, which necessarily takes some time.

At the end of the process, the lower graph is populated:

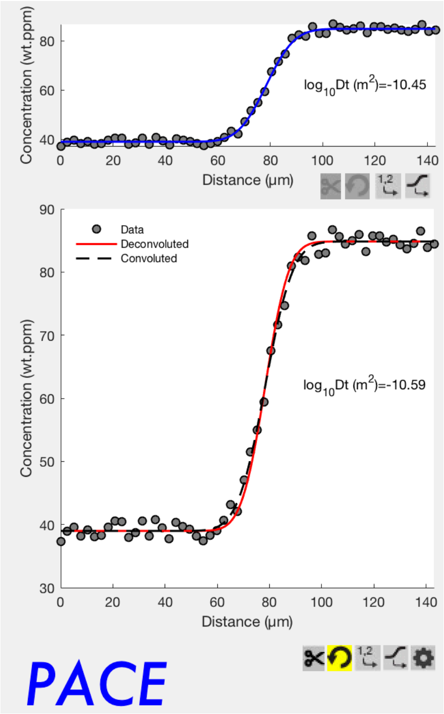

The lower graph shows the same scatter points as in the upper graph (with excluded data carried through, if applicable).
It also shows two curves – the solid red curve is PACE's best approximation of the true, deconvoluted profile, and the dashed black curve is the convoluted best fit to the data. Note that the dashed black curve only has the same form as the solid blue curve in the upper plot if the beam is Gaussian *(for more information on why this is the case, think about the relationship between the normal distribution and the error function, and look at (Ganguly et al., 1988)).*
The lower graph also presents a new value of logDt, which is the best estimate of the true logDt for these data. In this case, the difference is 0.1 log units.
The other change is that the cog icon at the lower right of the lower graph becomes enabled, as do the two exclusion icons (see Section 2.9), and the export buttons.

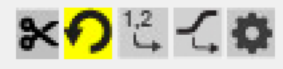

The data exclusion/reversion process for the lower graph functions in the same way as in Section 2.9. The cog icon toggles a second level of functionality, which allows near complete control over the figure appearance, including full export capabilities. This is discussed in more detail in Section 2.15.

2.13 Export from lower plot

As stated with regards to the top plot, many export options are available (detailed in section 2.15.8). The two icons below the lower plot:

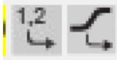

Are designed for quick export only. The functionality is very similar to the export buttons from the top plot. For example, if we have the plot:

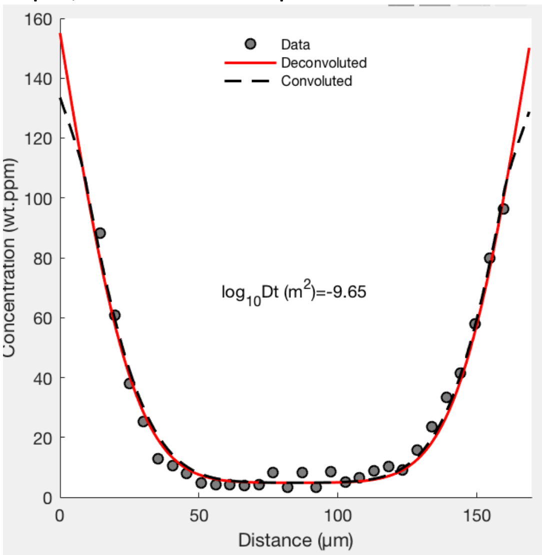

Then, upon pressing:

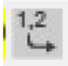

the fit parameters (C1, C2 and $log_{10}Dt$) are placed on the system clipboard. Then, they can be pasted into a spreadsheet or text program, e.g.:

	A	B
1	-9.649453	0.07699858
2	155.0756	13.47998
3	4.801496	2.327976

As above, pressing:

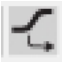

places the graph onto the system clipboard in vector format. Pasting the above figure into Adobe Illustrator gives:

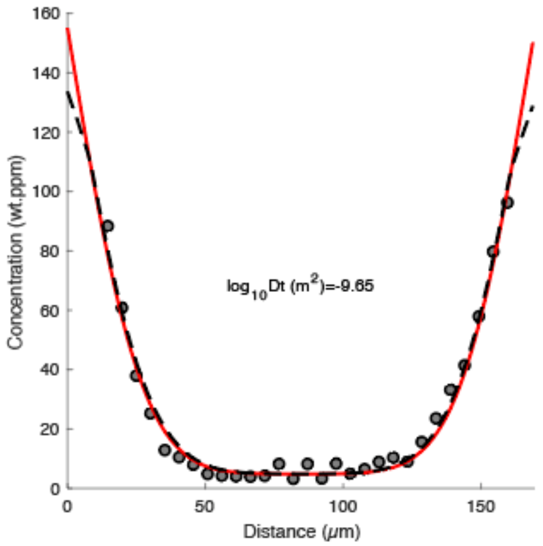

2.14 Dt, D t

The default output of PACE is $\log_{10}$Dt, i.e., the base 10 logarithm of the diffusion coefficient in m^2s^{-1} , multiplied by the time, in seconds, with units m^2. This allows the user to easily extract diffusion coefficients, if the time is known (e.g. experimental data), or extract times, if the diffusion coefficient is known (e.g. diffusion chronometry). Extracting $\log_{10}$D from $\log_{10}$Dt and $\log_{10}$t is done simply using:

$$log_{10}D = log_{10}Dt - log_{10}t$$

with the same formulation used for extracting t when D is known.
If the uncertainties are assumed to be symmetrical in $\log_{10}$ scale, then the uncertainties on time can be determined by propagating the uncertainties on $\log_{10}$D and $\log_{10}$Dt using:

$$\delta log_{10}t = \sqrt{(\delta log_{10}Dt)^2 + (\delta log_{10}D)^2}$$

The uncertainties on a given diffusion coefficient can be determined at any temperature (±pressure) using the uncertainties on the pre-exponential factor and activation energies (±activation volume). When calculating uncertainties at a given temperature, the covariance between the pre-exponential factor and the activation energy must be taken into account. A good description is given in Tirone et al. (2005)

2.15 Figure modification

PACE allows the lower figure to be modified and exported. The different options are relatively self-explanatory – only brief descriptions are given below.

Open the figure modification toggle by pressing the cog button:

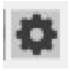

At this point, the window will enlarge to reveal a series of new tabs:

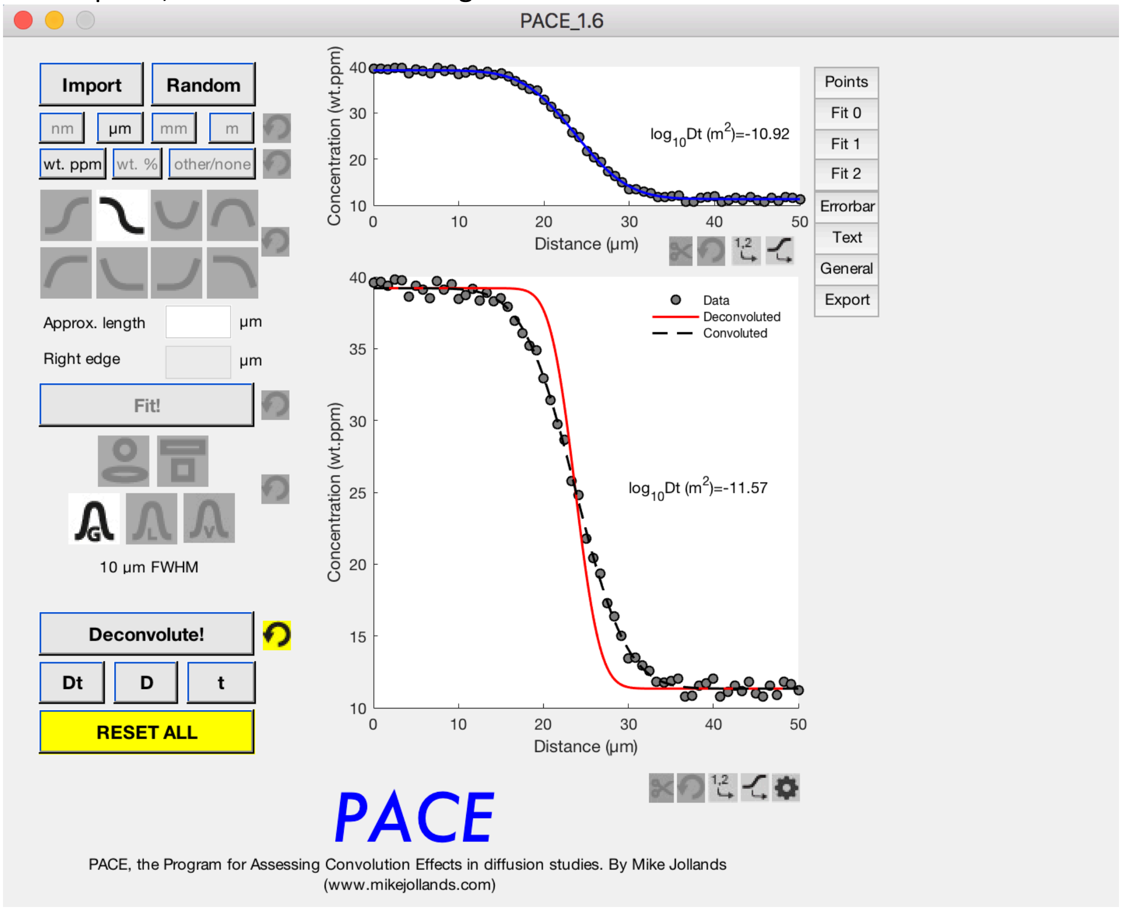

The seven buttons allow individual properties of the figure to be set:

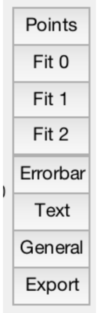

Pressing any of these will open a tab with different options, including colours, line widths, opacity, visibility etc.

2.15.1 Point modification

'Points' allows the point shape, size, fill colour, opacity, line width and line colour to be changed.

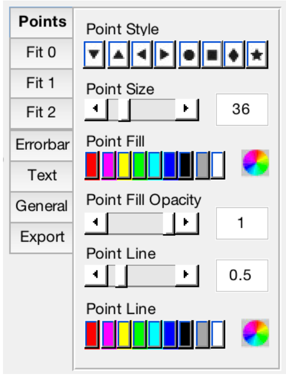

Clicking the colourwheel (in this tab, and all others) will open a new box:

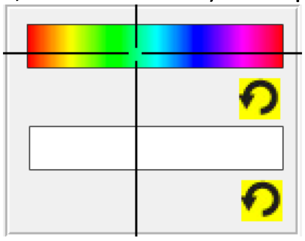

with a cursor. Click the first colourbar to select a colour, e.g. light green in the example above. This will open a second colourbar, allowing a shade of the same colour to be selected:

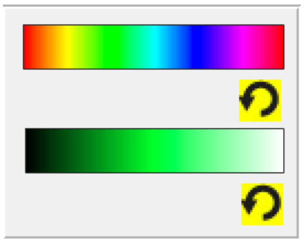

Here, dark green could be selected, for example.

2.15.2 Fit 0 modification

The curve labelled as 'Fit 0' is normally not shown. This is the fit presented in the top graph, without considering any deconvolution. This can be toggled on and off using the 'Visibility' checkbox.

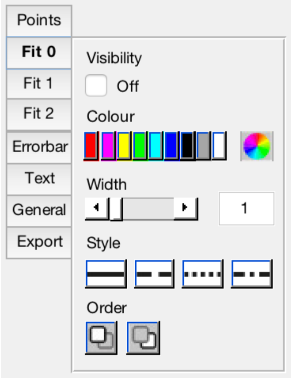

Toggling this 'Visible' will update the lower curve, e.g.:

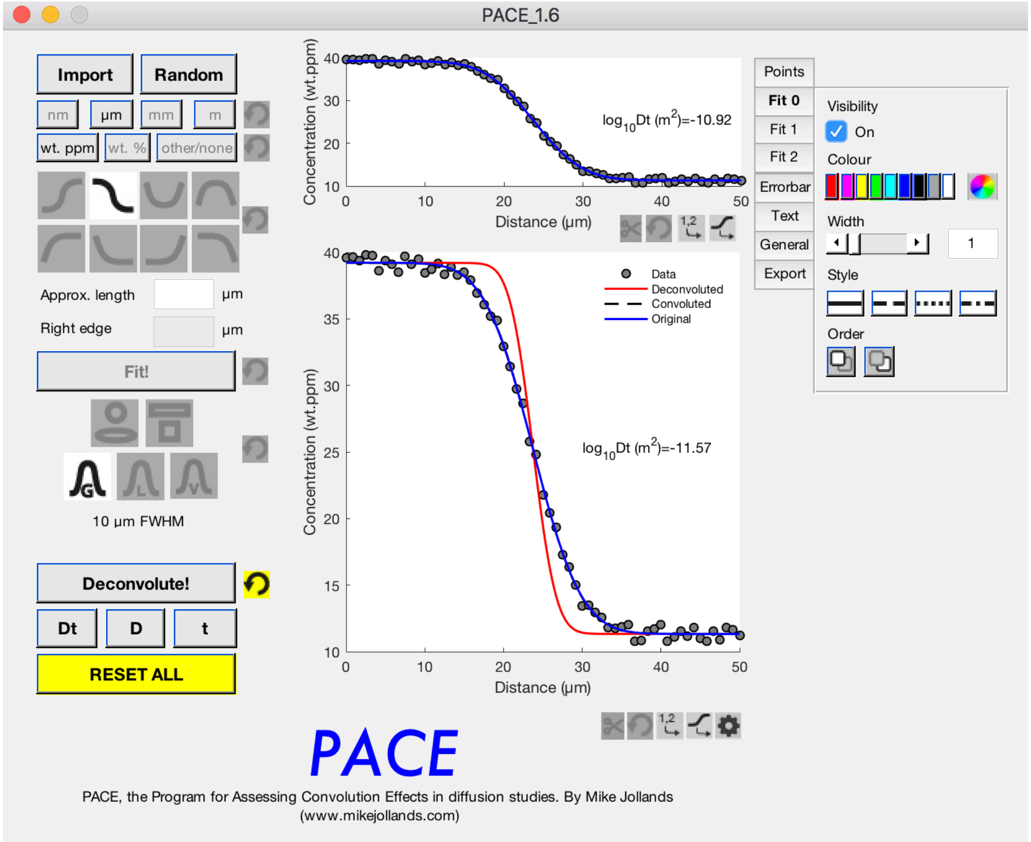

The line can be modified to change its width, style and order relative to the other lines and scatter points.

2.15.3 Fit 1 modification

'Fit 1' refers to the deconvoluted line. All options are the same as for fit 0

2.15.4 Fit 2 modification

'Fit 2' refers to the convoluted line. All options are the same as for fit 1 and fit 0.

2.15.5 Error bar modification

If the data does not include uncertainties, then all options in this window will be unavailable:

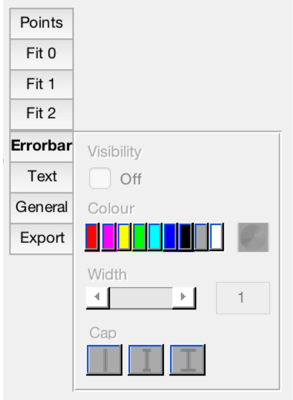

If uncertainties are available, then the options allow the error bars to be made invisible, change their widths, colour and cap size, similarly to the 'fit' options.

2.15.6 Text modification

This toggle allows various modifications to be made to the text on the lower plot.

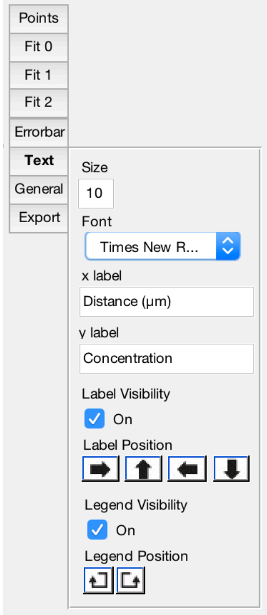

The text size, font, x and y axis labels can be modified. The 'Label Visibility' option removes or returns the default $\log_{10}$Dt… label on the graph, and this can be shifted up, down, left or right using the Label Position buttons. The visibility of the legend can be turned on and off, and the position of the legend can be moved clockwise or anticlockwise into pre-defined positions (north, south, southeast, southwest etc.).

2.15.7 General modification

The 'General' tab allows various other properties to be modified.

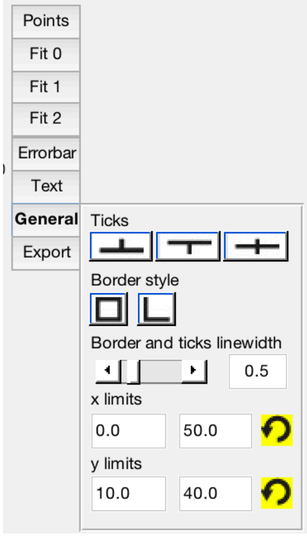

The ticks can be moved inside or outside of the axes, the border type can be changed as well as its linewidth, and the axes limits can be modified.

2.15.8 Export

The export tab allows export both of the lower graph, and the metadata, along with the actual data. This is useful if you want to take the data, curves, etc. and re-plot.

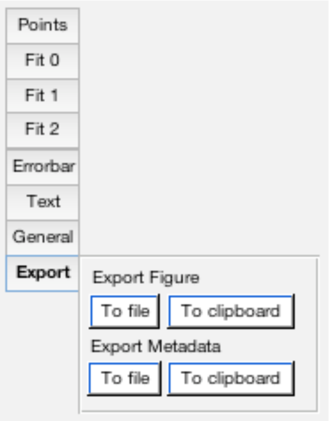

The top two buttons will export the figure either to a file (pdf, eps, svg, bmp, tiff, jpg options), or onto the clipboard. The clipboard option has exactly the same functionality as the export to clipboard button at the lower right corner of the lower plot.
The lower two buttons will export the data associated with the figure. The 'To file' button will open a dialog box where you can select the file type (csv, xlsx or txt). This will create a spreadsheet as follows, where the words in the left-most column describes the row contents, then the rest of the row gives the data:

	A	B	C	D	E	F	G	H	I	J	K
1	PACE Output	6/10/20 18:00									
2											
3	Distance, µm	0	2.74418605	5.48837209	8.23255814	10.9767442	13.7209302	16.4651163	19.2093023	21.9534884	24.6976744
4	Distance, m	0	2.74E-06	5.49E-06	8.23E-06	1.10E-05	1.37E-05	1.65E-05	1.92E-05	2.20E-05	2.47E-05
5	Concentratio	53.54793842	55.158901	56.0051567	56.3500276	54.5744566	54.399148	58.2857699	52.3402679	57.6559676	57.8559996
6											
7											
8	C1 (fit 0)	55.18809364	1.03473825								
9	C2 (fit 0)	107.0100627	1.01191565								
10	logDt (fit 0)	-10.24747217	0.10055103								
11	model distan	0	3.93E-07	7.87E-07	1.18E-06	1.57E-06	1.97E-06	2.36E-06	2.75E-06	3.15E-06	3.54E-06
12	model conce	55.18809484	55.1880951	55.1880954	55.1880959	55.1880964	55.188097	55.1880977	55.1880986	55.1880997	55.188101
13											
14	Beam Type	square/rectangle									
15	Width	20									
16											
17	C1 (fit 1)	55.24317415	1.03780854								
18	C2 (fit 1)	106.9740101	1.01543137								
19	logDt (fit 1)	-10.42553003	0.15324848								
20	model distan	0	6.70E-07	1.34E-06	2.01E-06	2.68E-06	3.35E-06	4.02E-06	4.69E-06	5.36E-06	6.03E-06
21	convoluted (	55.24317421	55.2431742	55.2431743	55.2431744	55.2431745	55.2431746	55.2431749	55.2431753	55.2431759	55.2431767
22	deconvolute	55.24317416	55.2431742	55.2431742	55.2431742	55.2431742	55.2431742	55.2431742	55.2431742	55.2431742	55.2431742
23											

**Row**	**Contents**
1	Date and time of export
3	Distance vector associated with the input data, in the input units
4	As 3, converted to metres
5	Concentration associated with the input data
6	Uncertainties associated with the input data, if applicable
8	First concentration associated with the first fit (from the top graph)
9	As 8, for the second concentration
10	logDt from the first fit
11	Modelled distance from the first fit, this is always a 300 point vector
12	As 11, concentrations
14	Type of beam

15	Size of beam
17	First concentration associated with the second fit (from the lower graph)
18	As 8, for the second concentration
19	logDt from the second fit
20	Modelled distance from the second fit
21	Modelled convoluted profile from the second fit
22	Modelled deconvoluted profile from the second fit.

The 'To clipboard' button will place some data onto the clipboard, this time columnwise. Pressing this button then pasting gives a matrix of NaNs (Not a Number), with some cells filled, e.g.

	A	B	C	D	E	F	G	H
1	0	60.48211	NaN	0	60.07	0	60.07	60.07
2	1.33333	60.22475	NaN	0.10667	60.07	0.32	60.07	60.07
3	2.66667	58.67538	NaN	0.21333	60.07	0.64	60.07	60.07
4	4	60.05438	NaN	3.20E-01	6.01E+01	9.60E-01	6.01E+01	6.01E+01
5	5.33333	59.67101	NaN	0.42667	60.07	1.28	60.07	60.07
6	6.66667	60.89152	NaN	0.53333	60.06999	1.6	60.07	60.07
7	8	59.97703	NaN	0.64	60.06999	1.92	60.07	60.07
8	9.33333	60.25213	NaN	0.74667	60.06999	2.24	60.07	60.07
9	10.66667	58.91094	NaN	0.85333	60.06999	2.56	60.07	60.07
10	12	57.8295	NaN	0.96	60.06999	2.88	60.07	60.07
11	13.33333	56.69901	NaN	1.07E+00	6.01E+01	3.20E+00	6.01E+01	6.01E+01
12	14.66667	50.71872	NaN	1.17333	60.06998	3.52	60.07	60.07
13	16	44.7731	NaN	1.28	60.06998	3.84	60.07	60.07
14	17.33333	37.01907	NaN	1.38667	60.06998	4.16	60.07	60.07
15	18.66667	29.01514	NaN	1.49333	60.06997	4.48	60.07	60.07
16	20	22.27197	NaN	1.6	60.06997	4.8	60.07	60.07
17	21.33333	16.00401	NaN	1.70667	60.06996	5.12	60.07	60.07
18	22.66667	9.91054	NaN	1.81333	60.06995	5.44	60.07	60.07
19	24	8.55196	NaN	1.92	60.06995	5.76	60.07	60.07
20	25.33333	7.82595	NaN	2.03E+00	6.01E+01	6.08E+00	6.01E+01	6.01E+01
21	26.66667	7.10187	NaN	2.13333	60.06993	6.4	60.07	60.07
22	28	7.60816	NaN	2.24	60.06991	6.72	60.07	60.07
23	29.33333	7.4893	NaN	2.34667	60.0699	7.04	60.06999	60.07
24	30.66667	6.57038	NaN	2.45333	60.06988	7.36	60.06998	60.07
25	32	6.94633	NaN	2.56	60.06986	7.68	60.06994	60.07
26	NaN	NaN	NaN	2.66667	60.06984	8	60.06983	60.07
27	NaN	NaN	NaN	2.77333	60.06981	8.32	60.06955	60.07
28	NaN	NaN	NaN	2.88	60.06978	8.64	60.06886	60.07
29	NaN	NaN	NaN	2.98667	60.06975	8.96	60.06728	60.07
30	NaN	NaN	NaN	3.09333	60.06971	9.28	60.06383	60.07
31	NaN	NaN	NaN	3.2	60.06966	9.6	60.0567	60.07

Column	Contents
A	Distance vector associated with the input data, in the input units
B	Concentration associated with the input data
C	Uncertainties associated with the input data, if applicable
D	Modelled distance from the first fit
E	First concentration associated with the first fit
F	Modelled distance from the second fit
G	Modelled convoluted profile from the second fit
H	Modelled deconvoluted profile from the second fit.

3 Setting initial (and boundary) conditions with PACE-IC

3.1 Methods in PACE-IC

3.2 Start screen

PACE-IC has mostly the same functionality as PACE, but it allows an import of the initial and boundary condition. PACE-IC assumes that the boundary conditions are fixed, i.e. that the points at the start and end of the initial condition vector represent the boundaries. Opening PACE-IC from the start screen gives the following:

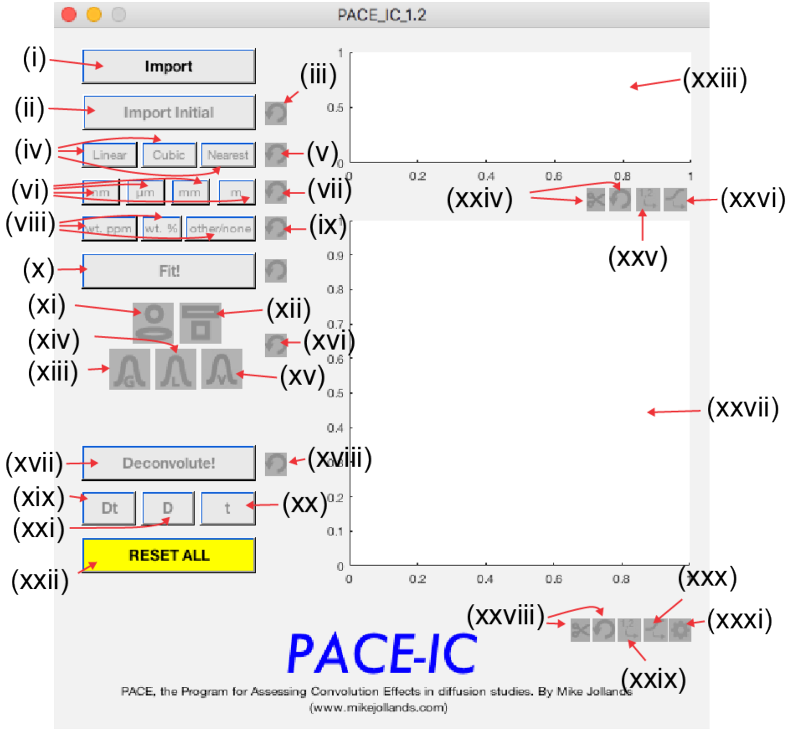

(i) Import Data (Section 3.3, p. 26)
(ii) Import Initial Condition (Section 3.4, p. 28)
(iii) Reset initial condition import
(iv) Interpolate the initial condition (Section 3.5, p. 30)
(v) Reset choice of initial condition
(vi) Select the distance units (Section 3.6, p. 31)
(vii) Reset the distance units
(viii) Select the concentration units (Section 3.7, p. 31)
(ix) Reset the concentration units

(v) Select the concentration units (Section 2.5, p. 8)
(vi) Reset the concentration units (Section 2.5, p. 8)
(vii) Sigmoidal curves (Section 2.6.1, p. 9)
(viii) U or upside-down-U shapes (Section 2.6.3, p. 10)
(ix) Half error function shapes (Section 2.6.2, p. 10)
(x) Reset shape choice
(xi) Add the approximate profile length (Section 2.7.1, p. 11)
(xii) Add the position of the right edge (if necessary/if desired) (Section 2.7.2, p. 11)
(xiii) Fit the data to the model (Section 2.8, p. 12)

(xiv) Reset the fit
(xv) Select a circular or elliptical beam shape (Section 2.11.2, p. 15)
(xvi) Select a square or rectangular beam (Section 2.11.1, p. 15)
(xvii) Select a Gaussian beam shape (Section 2.11.3, p. 15)
(xviii) Select a Lorentzian beam shape (Section 2.11.4, p. 15)
(xix) Select a Voigt (pseudo Voigt) shape (Section 2.11.5, p. 15)
(xx) Reset the curve shape
(xxi) Deconvolute the data (Section 2.12, p. 16)
(xxii) Reset the deconvolution
(xxiii) Output base 10 logarithm of the diffusion coefficient multiplied by time ($\log_{10}Dt$) – the default option. (Section 2.14, p. 18)
(xxiv) Output base 10 logarithm of the diffusion coefficient ($\log_{10}D$) (Section 2.14, p. 18)
(xxv) Output base 10 logarithm of time ($\log_{10}t$) (Section 2.14, p. 18)
(xxvi) Reset all – return to the start
(xxvii) Top axes, used for plotting the first fit.
(xxviii) Exclude data, and reset exclusions. (Section 2.9, p. 12)
(xxix) Simple export of fit parameters from first fit. (Section 2.10, p. 13)
(xxx) Simple export of top plot as vector graphic (Section 2.10, p. 13)
(xxxi) Second axis, for plotting deconvolution
(xxxii) Exclude data and reset exclusions from second plot
(xxxiii) Export fit data from lower plot (Section 2.13, p. 17)
(xxxiv) Export lower plot as vector graphic (Section 2.13, p. 17)
(xxxv) Toggle figure modification settings (Section 2.15, p. 19)

3.3 Import

Pressing import will open a standard import window, where any .csv, .xlsx, .xls or .txt file can be imported. Ideally, these should be files with only two columns, where the first column is distance (either from the interface, or along a profile) and the second is concentration. If this is not the case, a second dialog box will appear asking for extra input. **Warning: the Distance and Concentration columns must contain the same number of data points.**

Warning: in some complex Excel spreadsheets, there may be cells that appear empty, but are not (e.g. include a space). If such a cell exists somewhere in the same column as the data, PACE-IC will fail to import. This can be solved by copying and pasting the data to be imported into different columns, or, even better, into a new spreadsheet.

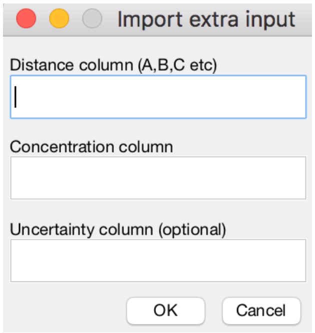

Use Excel-type notation for the columns, e.g. 1st column is A, 2nd is B, 27th is AA, etc:

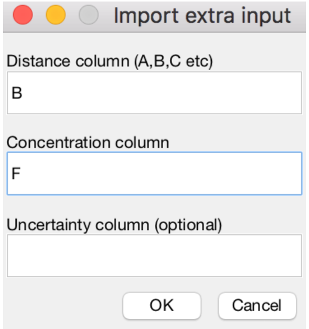

Pressing 'OK' will then import the data, 'Cancel' will return to the main screen.
Importing, or randomly generating data, will enable the next line of buttons.

Warning: PACE-IC may struggle if the units of distance are already in metres, for a micrometre/nanometre-long profile. It is better to keep the distance units in micrometres, rather than converting to metres before import.

In addition, uncertainties can be added, if desired, using the third box:

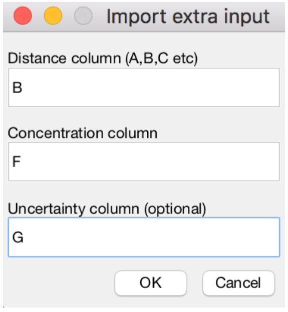

If uncertainties (e.g. σ) are added, they are converted to weights in PACE using $1/\sigma^2$. If weights are added, error bars will be added to the top right scatter plot
The imported data will then populate the top right plot:

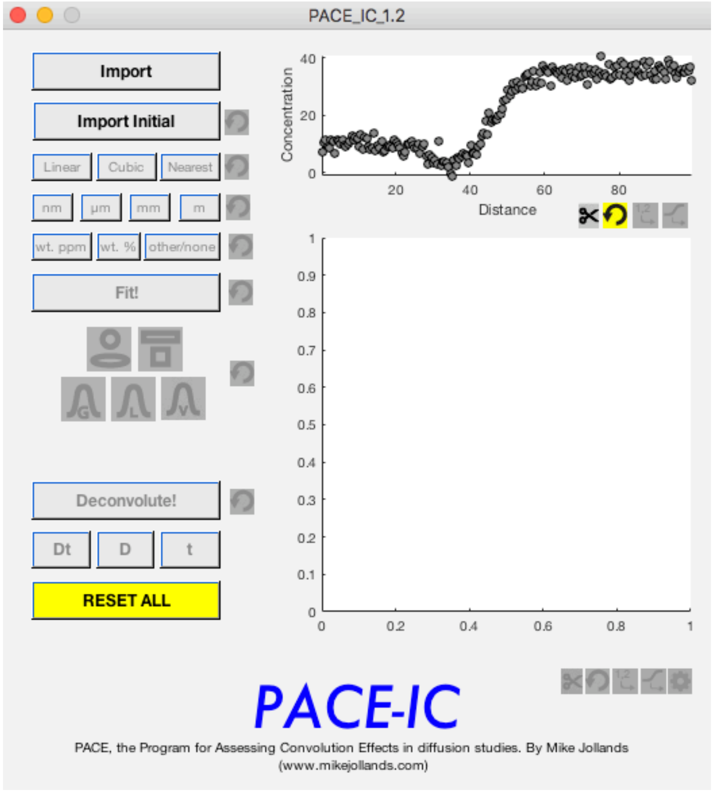

This then enables the next button, 'Import Initial'.

3.4 Import Initial condition

The initial condition that is imported needs to contain sufficient points to describe the whole initial profile, and the two boundary conditions. The initial condition is imported in the same way as the data: a standard 'open' dialogue box appears, from which the data can be selected. As with the data, the initial condition must be in two columns, with the first being distance, and the second being concentration. As above, if the imported data contains more than two columns, a window will appear asking for extra input:

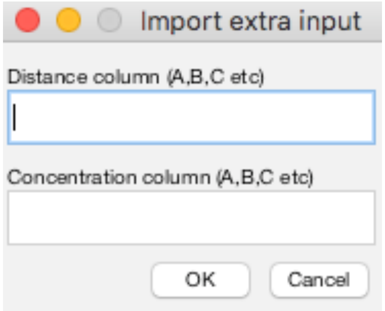

3.4.1 Formatting the initial condition

Important: 1) The initial condition vectors do not need to be the same length as the data.

2) The initial condition vectors need to have a wider range than (or the same range as) the data vector, which means that the lowest value in the distance column of the initial condition vector must be lower, or equal to, the lowest value in the distance column of the data vector. The highest value of the distance column of the initial condition vector

must be greater than, or equal to, the highest value of the distance column of the data vector.

3) The distance column of the initial condition vector cannot have any duplicate values.

For example, consider a case where we want to simulate a step function, with extremities at 0 and 300, with the step at 140. The value between 0 and 140 is 10, and the value between 140 and 300 is 50. As stated above, the distance column cannot have any duplicates, which means we must define the step using two values close to, but not exactly, the same, such as 139.9999 and 140. A spreadsheet with the initial conditions would look like this:

	A	B
1	0	10
2	139.9999	10
3	140	50
4	300	50
5		

PACE-IC will assume that the concentration at position 0 is always 10, and the concentration at position 300 is always 50.
The minimum number of points required to describe this step is four. If extra points are added, the outcome will not be changed. For example:

	A	B
1	0	10
2	30	10
3	60	10
4	90	10
5	120	10
6	139.9999	10
7	140	50
8	200	50
9	250	50
10	300	50

will give exactly the same result.

For a second example, let's assume we want the initial conditions to be a homogeneous zero concentration with length 300, with interfaces at positions 0 and 300 with concentrations of 10. Again, because duplicates are not permitted, a potential input could be:

	A	B
1	0	10
2	0.0001	0
3	299.999	0
4	300	10

Because PACE-IC uses a numerical method, any initial condition can be used.

3.4.2 Top graph following import

Once the initial data has been imported, the individual points will appear as red crosses on the top graph. In the following, the initial condition is:

	A	B
1	0.1	11
2	30	11
3	30.001	1
4	46	1
5	46.001	36
6	100	36

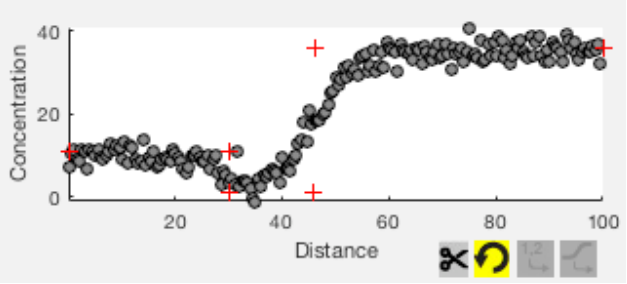

If, instead, we import a vector with uniform 0.1 spacing, we get:

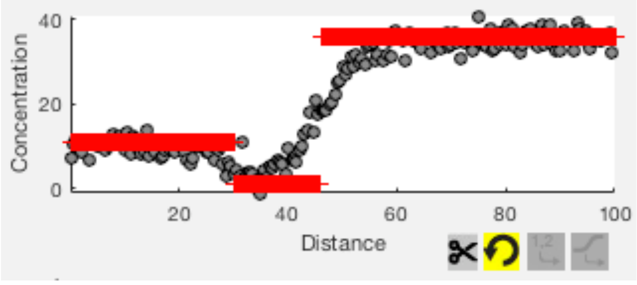

Again, the results should be identical (depending on the interpolation method).
Importing the data leads to the 'Import Initial' button being disabled, and the next buttons becoming enabled:

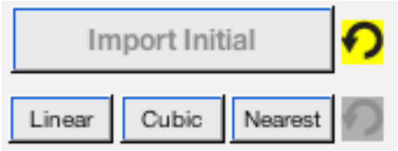

3.5 Interpolating the initial condition

Once the initial condition is imported, it is interpolated. Generally, the recommended interpolation method is linear, but the other methods will be useful in specific circumstances. Once an interpolation method is selected, the red crosses will disappear, and will be replaced by a dashed red line. Following selection of one interpolation method, the others will be disabled, and the next line of buttons will be enabled.

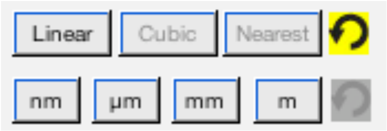

3.5.1 Linear Interpolation

This will interpolate by drawing straight lines between every grid point. For the above example (with fewer points), this gives:

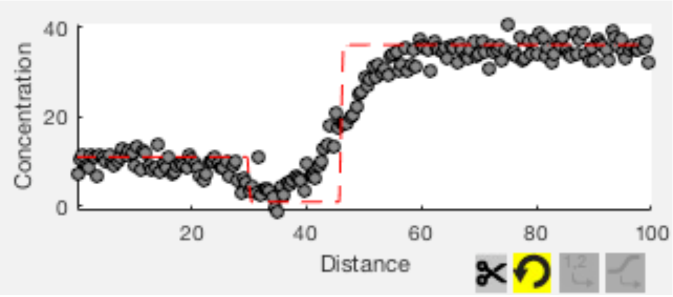

3.5.2 Cubic interpolation

In the case shown here, this will give identical results to the linear interpolation. However, it will give different results for curved initial conditions, for example.

3.5.3 Nearest Interpolation

This will interpolate based on the nearest neighbour. Again, in this case, the results are identical.

3.6 Select Distance Units

The available units are nanometres (nm), micrometres (μm), millimetres (mm) and metres (m), assuming that most geologically-relevant measurements will be in either nm or μm.

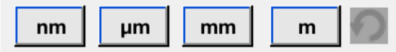

Selecting one of these will cause the others to become disabled and illuminate the reset button on the right, and the next line of options:

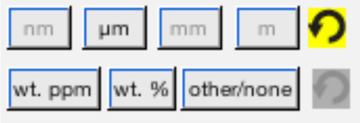

Additionally, the selection will update the x-axis of the top-right graph:

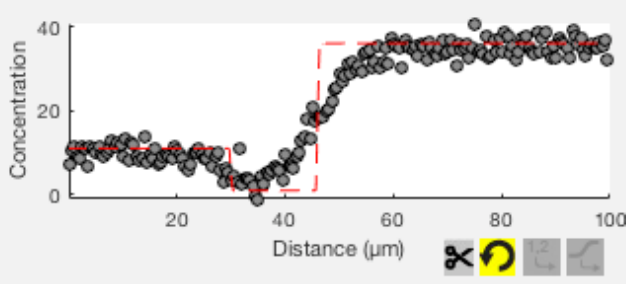

and some other text through the program.

3.7 Select Concentration Units

After selecting distance units, the buttons allowing concentration units to be selected becomes enabled:

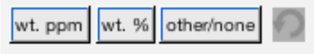

Select one unit. The assumption is that most relevant data will be in wt. ppm. Upon selecting the unit, the graph will update its y-axis:

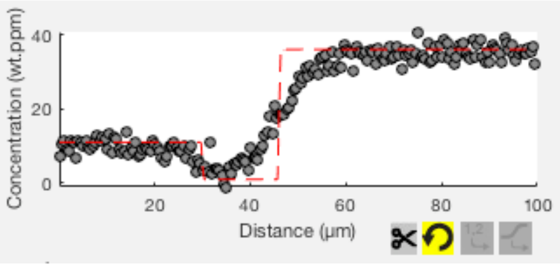

the other options will become disabled and the relevant reset button, as well as the next button, becomes enabled:

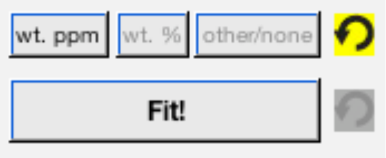

Good to know: given the solutions of the diffusion equation used in PACE-IC, this step *doesn't matter*. This is because PACE-IC assumes diffusion is concentration independent. The step is added simply to have a unit on the y-axis, and because there is a plan to include concentration-dependence in a future version of PACE and PACE-IC.

3.8 Fit button

The first fit can be done by pressing the Fit button:

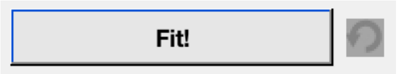

After pressing fit, a curve will appear over the data on the top right graph. The appearance of this cannot be modified. In addition, the value of Dt will be presented as a base 10 logarithm in the plot:

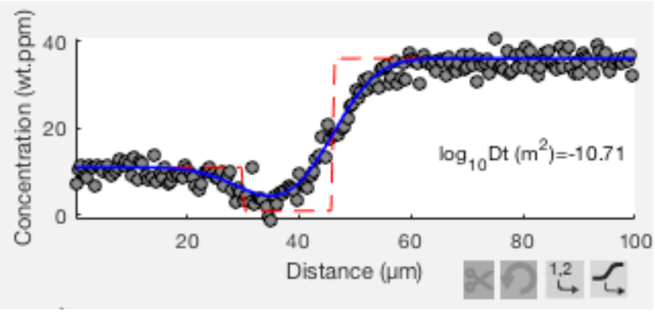

This fitting is done by non-linear least squares regression, using the lsqnonlin solver in MATLAB. Weighting is added if required.
Pressing the reset button next to the 'Fit!' button will remove the fitted curve from the top right plot.
This then enables the next five buttons, and disables the fit button:

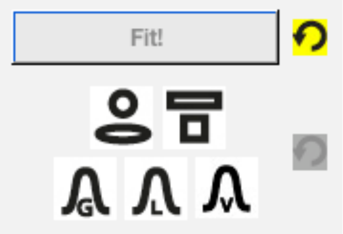

3.9 Selecting beam type

After fitting, the five icons showing beam shapes become available. The shapes supported by PACE-IC are circular/elliptical, square/rectangular, Gaussian, Lorentzian and Voigt.

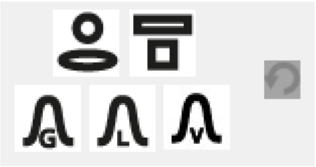

Upon selecting one of the beam types, the others become unavailable and reset becomes illuminated. As before, the choice can then be repeated using the reset button.

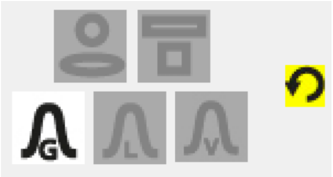

When any button is pressed, a dialog box opens, asking for further input. This is where you tell PACE-IC the beam size. PACE-GD may help to determine this value, where it is not known.

3.9.1 Square/rectangular

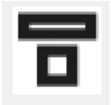

For square/rectangular beams, the width is required:

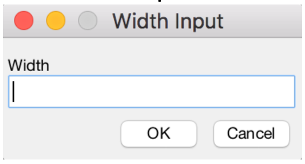

3.9.2 Circular/elliptical

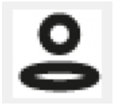

For the circular/elliptical beams, the diameter is needed

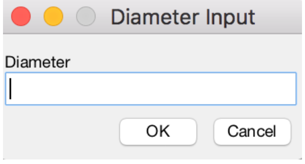

3.9.3 Gaussian

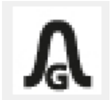

For Gaussian beams, the full width at half maximum (FWHM) is needed:

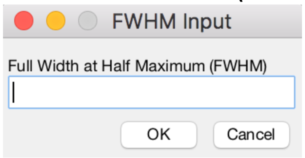

3.9.4 Lorentzian

The requirement is the same as for Gaussian beams (the FWHM)

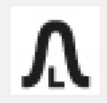

3.9.5 Pseudo-Voigt

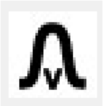

For beams best approximated by pseudo-Voigt shapes, the FWHM of both the Gaussian and Lorentian components is needed:

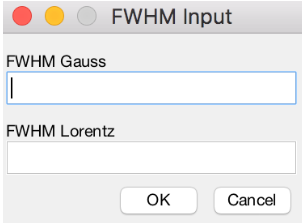

3.9.6 Input reminder

Once the beam size is given, text appears below the beam shape selection icons reminding the user of the inputted value, e.g.:

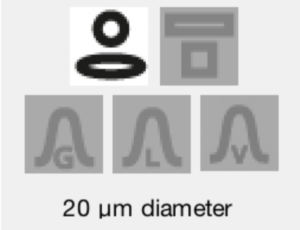

3.10 Deconvolution

The main functionality of PACE is contained in the 'Deconvolute' button. This refits the data using the given beam type and size, and gives an estimate of the true (***deconvoluted***) diffusion profile, along with the best fit to the measured (***convoluted***) profile. The nomenclature basically assumes that all measured profiles are convoluted to some degree, which is either negligible, or important.

Upon pressing deconvolute:

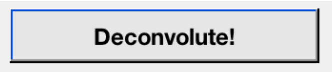

a wait-bar appears:

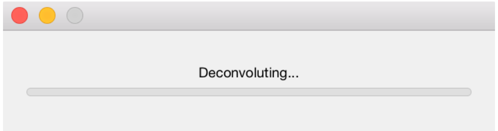

The wait bar normally takes ~1-2 seconds to complete. The delay is because PACE employs a multi-start solver to maximise the chances of obtaining a global minimum, which necessarily takes some time.

At the end of the process, the lower graph is populated:

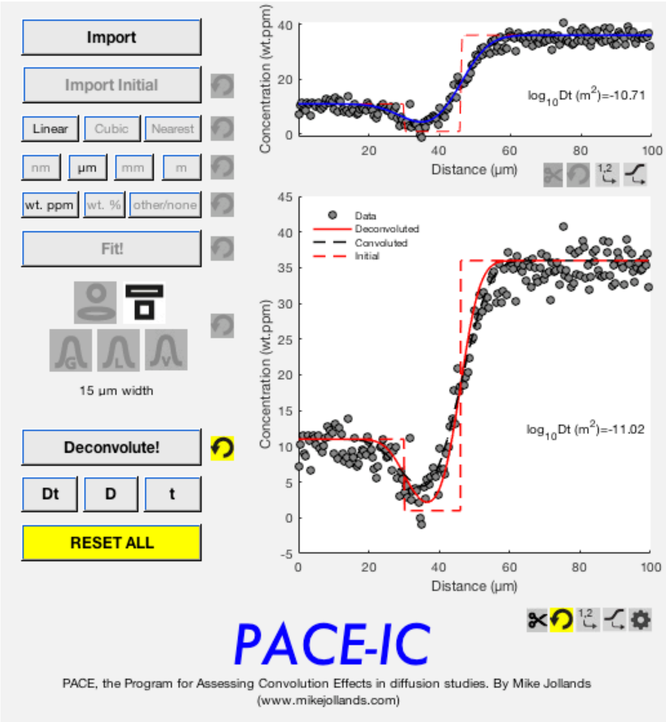

The lower graph shows the same scatter points as in the upper graph (with excluded data carried through, if applicable).

It also shows two curves – the solid red curve is PACE-IC's best approximation of the true, deconvoluted profile, and the dashed black curve is the convoluted best fit to the data.

The lower graph also presents a new value of logDt, which is the best estimate of the true logDt for these data. In this case, the difference is 0.3 log units.

The other change is that the cog icon at the lower right of the lower graph becomes enabled, as do the two exclusion icons (section 2.9), and the export buttons.

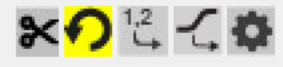

The data exclusion/reversion process for the lower graph functions in the same way as in PACE (section 2.9). The cog icon toggles a second level of functionality, which allows near complete control over the figure appearance, including full export capabilities. This is discussed in more detail in section 2.15.

3.11 Export from lower plot

As stated with regards to the top plot, many export options are available (detailed in section 3.13.8). The two icons below the lower plot:

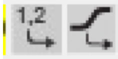

Are designed for quick export only. The functionality is very similar to the export buttons from the top plot. For example, if we have the plot:

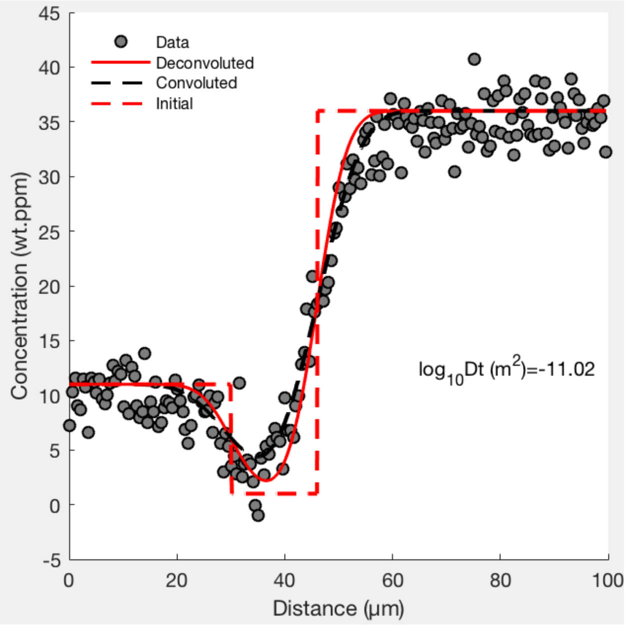

Then, upon pressing:

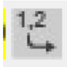

the fit parameter ($\log_{10}$Dt) are placed on the system clipboard, along with uncertainty. Then, they can be pasted into a spreadsheet or text program, e.g.:

	A	B
1	-11.0218	0.145827

As above, pressing:

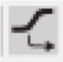

places the graph onto the system clipboard in vector format. Pasting the above figure into Adobe Illustrator gives:

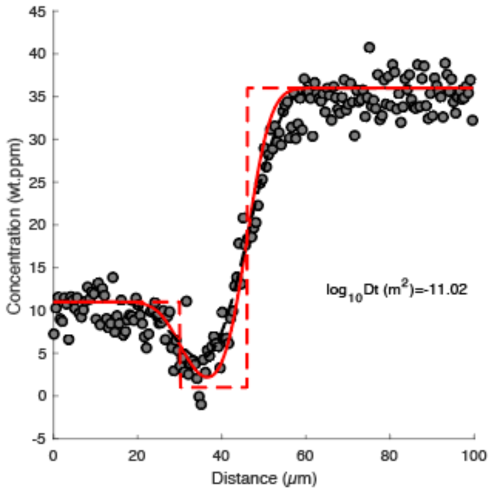

3.12 Dt, D t

The default output of PACE is $\log_{10}$Dt, i.e., the base 10 logarithm of the diffusion coefficient in m^2s^{-1} , multiplied by the time, in seconds, with units m^2. This allows the user to easily extract diffusion coefficients, if the time is known (e.g. experimental data), or extract times,

if the diffusion coefficient is known (e.g. diffusion chronometry). Extracting $log_{10}D$ from $log_{10}Dt$ and $log_{10}t$ is done simply using:

$$log_{10}D = log_{10}Dt - log_{10}t$$

with the same formulation used for extracting t when D is known.
If the uncertainties are assumed to be symmetrical in log_{10} scale, then the uncertainties on time can be determined by propagating the uncertainties on $log_{10}D$ and $log_{10}Dt$ using:

$$\delta log_{10}t = \sqrt{(\delta log_{10}Dt)^2 + (\delta log_{10}D)^2}$$

The uncertainties on a given diffusion coefficient can be determined at any temperature (±pressure) using the uncertainties on the pre-exponential factor and activation energies (±activation volume). When calculating uncertainties at a given temperature, the covariance between the pre-exponential factor and the activation energy must be taken into account. A good description is given in Tirone et al. (2005)

3.13 Figure modification

PACE allows the lower figure to be modified and exported. The different options are relatively self-explanatory – only brief descriptions are given below.
Open the figure modification toggle by pressing the cog button:

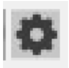

At this point, the window will enlarge to reveal a series of new tabs:

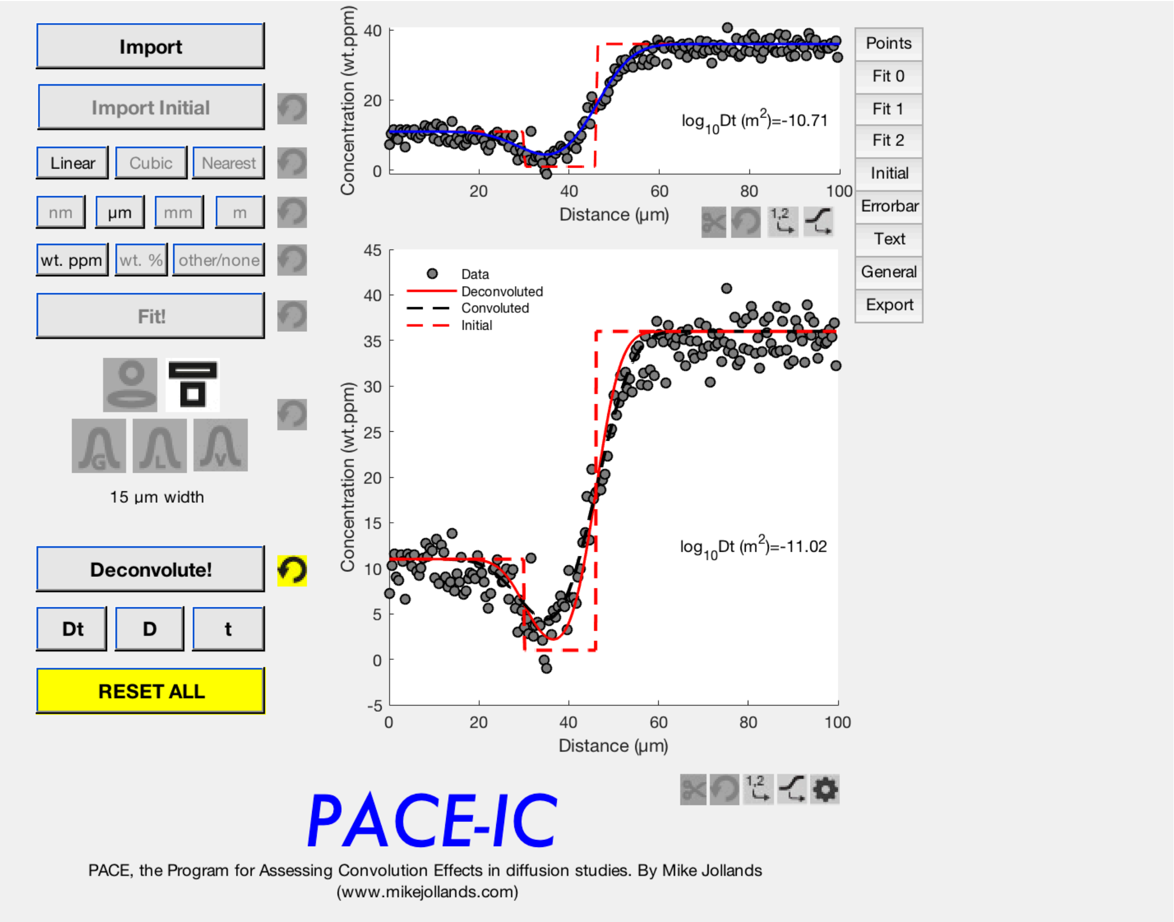

The nine buttons allow individual properties of the figure to be set:

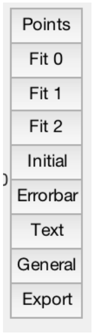

Pressing any of these will open a tab with different options, including colours, line widths, opacity, visibility etc.

3.13.1 Point modification

'Points' allows the point shape, size, fill colour, opacity, line width and line colour to be changed.

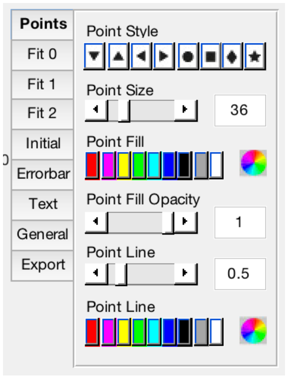

Clicking the colourwheel (in this tab, and all others) will open a new box:

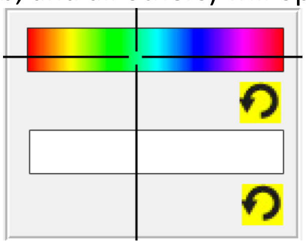

with a cursor. Click the first colourbar to select a colour, e.g. light green in the example above. This will open a second colourbar, allowing a shade of the same colour to be selected:

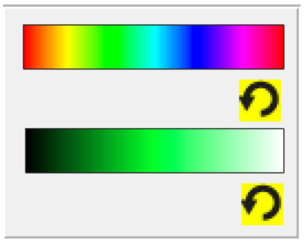

Here, dark green could be selected, for example.

3.13.2 Fit 0 modification

The curve labelled as ‘Fit 0’ is normally not shown. This is the fit presented in the top graph, without considering any deconvolution. This can be toggled on and off using the ‘Visibility’ checkbox.

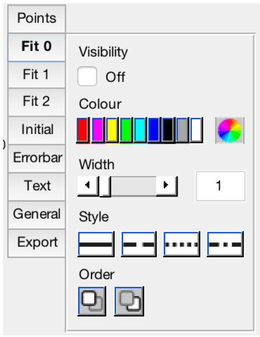

Toggling this ‘Visible’ will update the lower curve, e.g.:

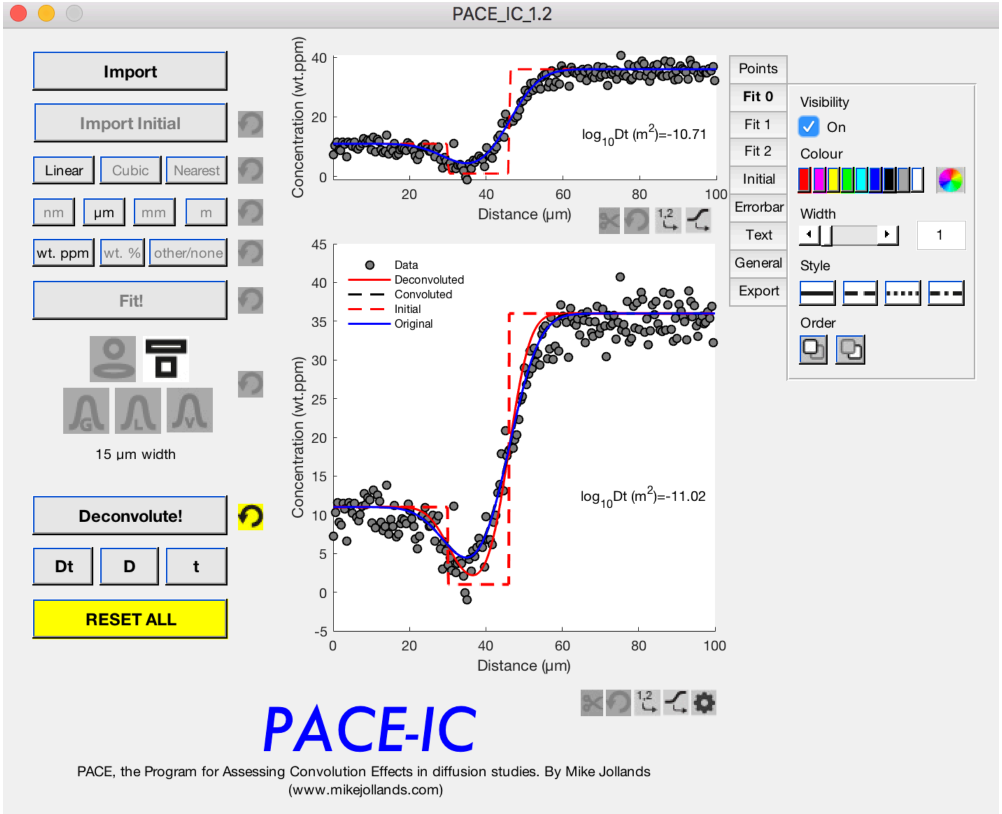

The line can be modified to change its width, style and order relative to the other lines and scatter points.

3.13.3 Fit 1 modification

‘Fit 1’ refers to the deconvoluted line. All options are the same as for fit 0

3.13.4 Fit 2 modification

‘Fit 2’ refers to the convoluted line. All options are the same as for fit 1 and fit 0.

3.13.5 Error bar modification

If the data does not include uncertainties, then all options in this window will be unavailable:

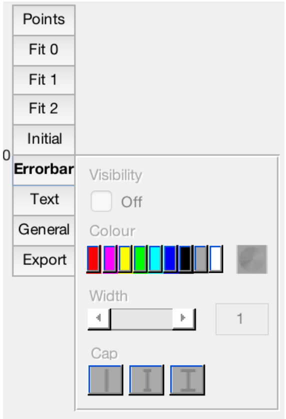

If uncertainties are available, then the options allow the error bars to be made invisible, change their widths, colour and cap size, similarly to the 'fit' options.

3.13.6 Text modification

This toggle allows various modifications to be made to the text on the lower plot.

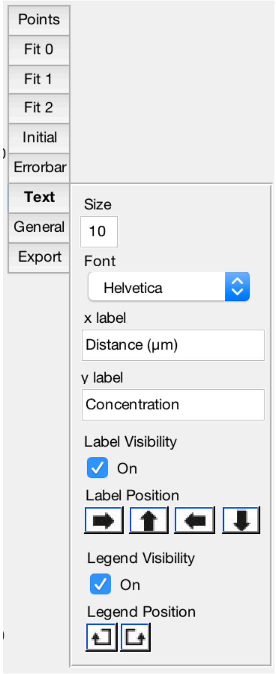

The text size, font, x and y axis labels can be modified. The ‘Label Visibility’ option removes or returns the default $\log_{10}$Dt… label on the graph, and this can be shifted up, down, left or right using the Label Position buttons. The visibility of the legend can be turned on and off, and the position of the legend can be moved clockwise or anticlockwise into pre-defined positions (north, south, southeast, southwest etc.).

3.13.7 General modification

The ‘General’ tab allows various other properties to be modified.

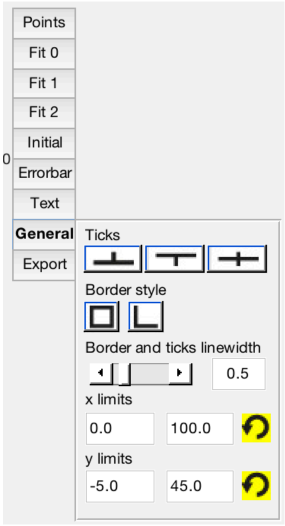

The ticks can be moved inside or outside of the axes, the border type can be changed as well as its linewidth, and the axes limits can be modified.

3.13.8 Export

The export tab allows export both of the lower graph, and the metadata, along with the actual data. This is useful if you want to take the data, curves, etc. and re-plot.

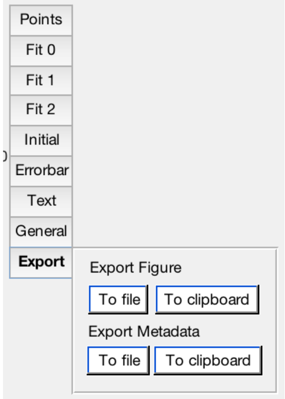

The top two buttons will export the figure either to a file (pdf, eps, svg, bmp, tiff, jpg options), or onto the clipboard. The clipboard option has exactly the same functionality as the export to clipboard button at the lower right corner of the lower plot.
The lower two buttons will export the data associated with the figure. The 'To file' button will open a dialog box where you can select the file type (csv, xlsx or txt). This will create a spreadsheet as follows, where the words in the left-most column describes the row contents, then the rest of the row gives the data:

	A	B	C	D	E	F	G	H	I	
1	PACE Output	6/11/20 19:23								
2										
3	Distance, µm	0.1	0.6	1.1	1.6	2.1	2.6	3.1	3.6	
4	Distance, m	1.00E-07	6.00E-07	1.10E-06	1.60E-06	2.10E-06	2.60E-06	3.10E-06	3.60E-06	
5	Concentratic	7.240545052	10.2668575	11.5268596	8.99181097	8.71338915	11.4705977	10.7514739	6.61059539	1
6										
7	Initial Condit	0.1	30	30.1	46	46.1	100			
8	Initial Condit	11	11	1	1	36	36			
9										
10	logDt (fit 0)	-10.70753915	0.06895856							
11	model distan	1.00E-07	1.10E-06	2.10E-06	3.10E-06	4.10E-06	5.10E-06	6.09E-06	7.09E-06	
12	model conce	11	10.9999872	10.9999662	10.9999248	10.9998412	10.9996755	10.9993564	10.9987588	
13	model initial	11	11	11	11	11	11	11	11	
14										
15	Beam Type	square/rectangle								
16	Width	15								
17										
18										
19	logDt (fit 1)	-11.02175797	0.14582728							
20	model distan	1.00E-07	2.24E-07	3.49E-07	4.73E-07	5.98E-07	7.22E-07	8.46E-07	9.71E-07	
21	convoluted (	10.99999993	10.9999999	10.9999999	10.9999999	10.9999999	10.9999998	10.9999998	10.9999998	1
22	deconvolute	11	11	11	11	11	11	11	11	
23	initial conditi	11	11	11	11	11	11	11	11	

**Row**	**Contents**
1	Date and time of export
3	Distance vector associated with the input data, in the input units
4	As 3, converted to metres
5	Concentration associated with the input data
6	Uncertainties associated with the input data, if applicable
8	Initial condition distance points
9	Initial condition concentrations
10	logDt from the first fit
11	Modelled distance from the first fit, this is always a 300 point vector
12	As 11, concentrations
13	As 12, initial concentrations
15	Type of beam
16	Size of beam
19	logDt from the second fit
20	Modelled distance from the second fit
21	Modelled convoluted profile from the second fit
22	Modelled deconvoluted profile from the second fit.
23	Modelled initial condition from the second fit.

The 'To clipboard' button will place some data onto the clipboard, this time columnwise. Pressing this button then pasting gives a matrix of NaNs (Not a Number), with some cells filled, e.g.

	A	B	C	D	E	F	G	H	I
1	0.10	7.24		0.10	11.00	0.10	11.00	11.00	11.00
2	0.60	10.27		1.10	11.00	0.22	11.00	11.00	11.00
3	1.10	11.53		2.10	11.00	0.35	11.00	11.00	11.00
4	1.60	8.99		3.10	11.00	0.47	11.00	11.00	11.00
5	2.10	8.71		4.10	11.00	0.60	11.00	11.00	11.00
6	2.60	11.47		5.10	11.00	0.72	11.00	11.00	11.00
7	3.10	10.75		6.09	11.00	0.85	11.00	11.00	11.00
8	3.60	6.61		7.09	11.00	0.97	11.00	11.00	11.00
9	4.10	11.58		8.09	11.00	1.10	11.00	11.00	11.00
10	4.60	11.18		9.09	11.00	1.22	11.00	11.00	11.00
11	5.10	10.23		10.09	10.99	1.34	11.00	11.00	11.00
12	5.60	11.49		11.09	10.99	1.47	11.00	11.00	11.00
13	6.10	9.66		12.09	10.98	1.59	11.00	11.00	11.00
14	6.60	9.22		13.09	10.96	1.72	11.00	11.00	11.00
15	7.10	10.07		14.09	10.94	1.84	11.00	11.00	11.00
16	7.60	11.10		15.09	10.91	1.97	11.00	11.00	11.00
17	8.10	12.73		16.08	10.86	2.09	11.00	11.00	11.00
18	8.60	11.32		17.08	10.79	2.21	11.00	11.00	11.00
19	9.10	12.19		18.08	10.70	2.34	11.00	11.00	11.00
20	9.60	11.94		19.08	10.57	2.46	11.00	11.00	11.00
21	10.10	8.97		20.08	10.41	2.59	11.00	11.00	11.00
22	10.60	13.23		21.08	10.19	2.71	11.00	11.00	11.00
23	11.10	8.32		22.08	9.93	2.84	11.00	11.00	11.00
24	11.60	12.57		23.08	9.61	2.96	11.00	11.00	11.00
25	12.10	11.74		24.08	9.23	3.09	11.00	11.00	11.00
26	12.60	9.03		25.08	8.79	3.21	11.00	11.00	11.00
27	13.10	7.97		26.07	8.29	3.33	11.00	11.00	11.00
28	13.60	8.47		27.07	7.76	3.46	11.00	11.00	11.00
29	14.10	13.86		28.07	7.20	3.58	11.00	11.00	11.00
30	14.60	7.46		29.07	6.62	3.71	11.00	11.00	11.00
31	15.10	9.36		30.07	6.06	3.83	11.00	11.00	11.00
[illegible]	[illegible]	[illegible]		[illegible]	[illegible]	[illegible]	[illegible]	[illegible]	[illegible]

**Column**	**Contents**
A	Distance vector associated with the input data, in the input units
B	Concentration associated with the input data
C	Uncertainties associated with the input data, if applicable
D	Modelled distance from the first fit
E	First concentration associated with the first fit
F	Modelled distance from the second fit
G	Modelled convoluted profile from the second fit
H	Modelled deconvoluted profile from the second fit.
I	Initial condition from the second fit.

4 Determining beam size with PACE-GD

Depending on the nature of the beam, measuring the beam size can be done in several ways. For square/rectangular/circular/elliptical beams, the size can be measured after analysis from photomicrographs using freeware such as FIJI/ImageJ (Schindelin et al., 2015). For flat-bottomed, ~vertically sided pits, the relevant diameter/width is that of the top of the pit. For other beam types, the best practice is to measure, or simulate a measurement

of, a step function. A curve is then fitted to the resulting function, which will be smoothed out to some degree, which gives the beam size.
PACE-GD does this all for you, with the methods presented in the accompanying manuscript.
Firstly, open PACE-GD. The layout is effectively a much simpler version of PACE.

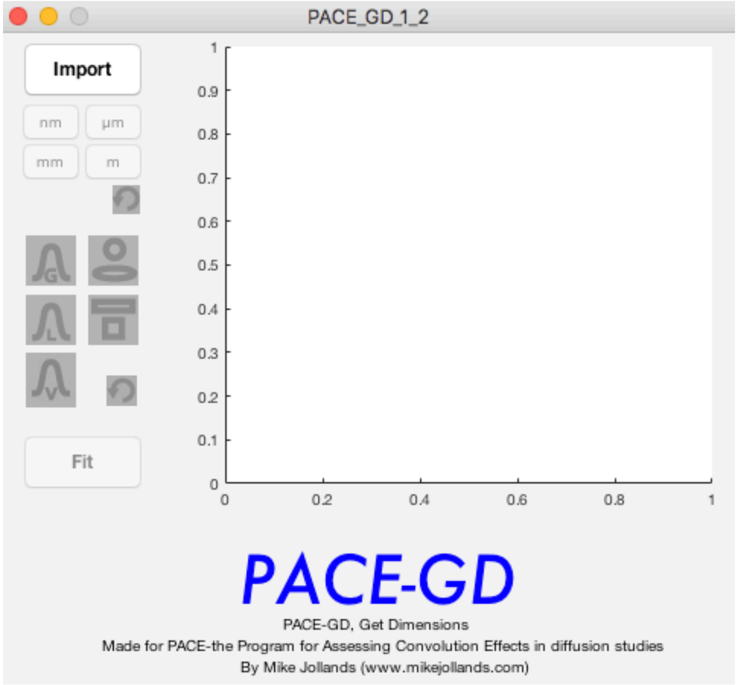

Then, import some data. This works in the same way as the PACE import – if the imported file (csv, xls, xlsx, txt) has more than two columns, you will be prompted to tell PACE-GD which column the distance and concentration vectors are in.
Shown below is greyscale data taken from a cathodoluminescence image of a Ti in quartz step function (extracted in imageJ).

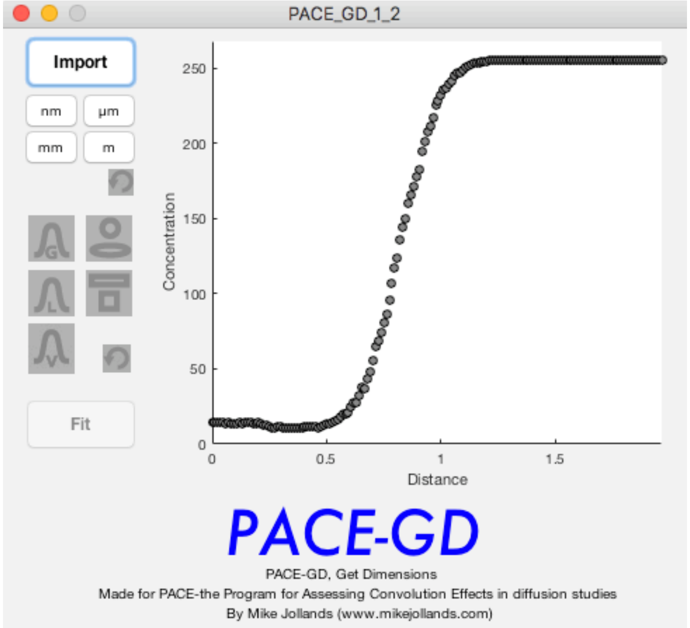

First, select the distance units:

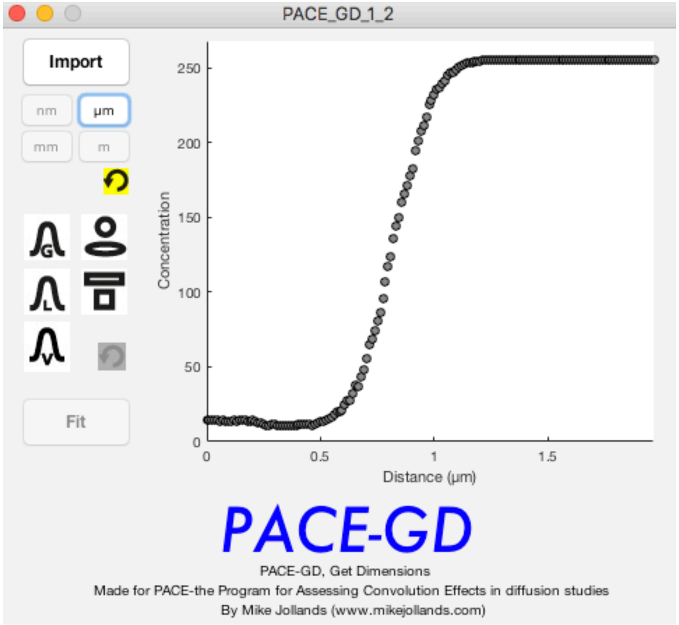

Then, simply select the beam type and press fit. Cathodoluminescence should give a Gaussian type interaction, so the G is selected (see Section 2.11 above). Then press 'Fit', which gives:

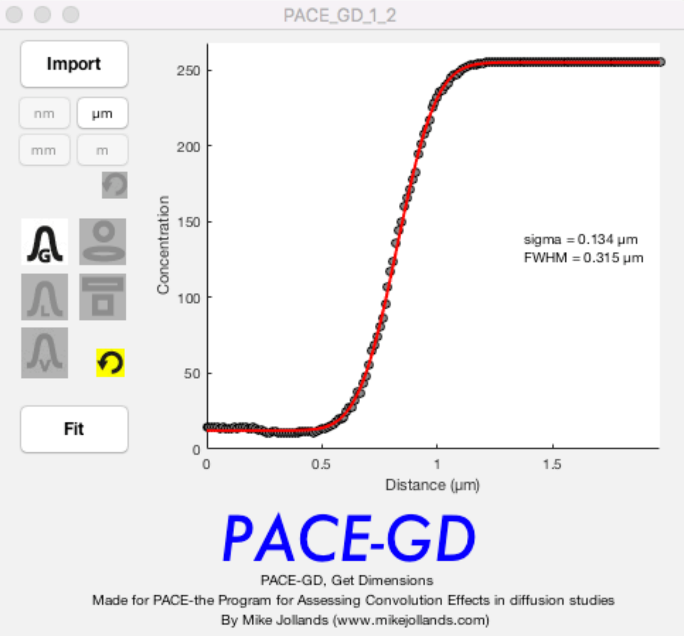

For a Gaussian type beam, one sigma and the FWHM are presented. Because a pseudo-Voigt shape is made of Gaussian and Lorentzian components, the V button can also be used, giving:

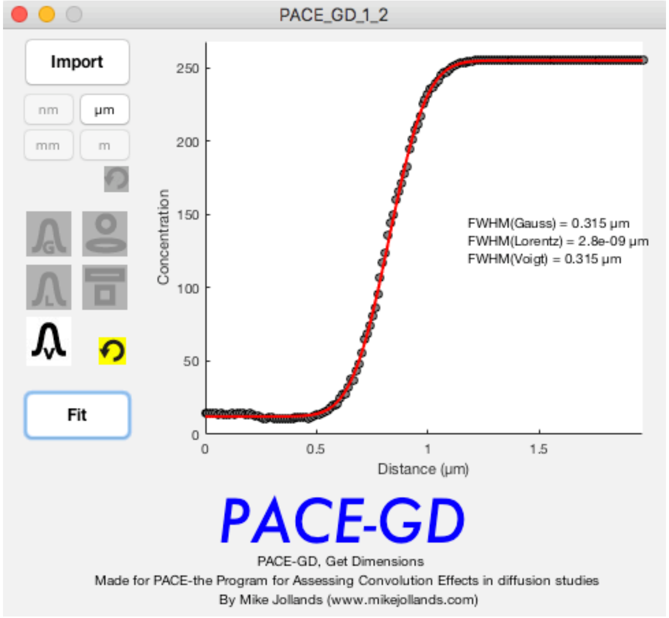

In this case, the FWHM of the Gaussian component is the same as in the previous fit, and the FWHM of the Lorentzian component is effectively zero. This confirms that the beam interaction is purely Gaussian, and the Lorentzian component is negligible.

The FWHM(s)/diameter/width determined using PACE-GD can then be used directly in PACE.

As in PACE, the steps can be undone using the yellow arrows:

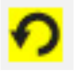

5 References

Browning, G. num2clip: copy numerical arrays to clipboard (https://www.mathworks.com/matlabcentral/fileexchange/8472-num2clip-copy-numerical-arrays-to-clipboard), MATLAB Central File Exchange. Retrieved January 3, 2020.

Carslaw, H. S., and Jaeger, J. C., 1959, Conduction of heat in solids.

Chakraborty, S., 1997, Rates and mechanisms of Fe-Mg interdiffusion in olivine at 980°-1300°C: Journal of Geophysical Research: Solid Earth, v. 102, no. B6, p. 12317-12331.

Chakraborty, S., and Ganguly, J., 1992, Cation Diffusion in Aluminosilicate Garnets - Experimental-Determination in Spessartine-Almandine Diffusion Couples, Evaluation of Effective Binary Diffusion-Coefficients, and Applications: Contributions to Mineralogy and Petrology, v. 111, no. 1, p. 74-86.

Crank, J., 1975, The Mathematics of Diffusion, Oxford, Oxford University Press.

Crispin, K. L., and Van Orman, J. A., 2010, Influence of the crystal field effect on chemical transport in Earth's mantle: Cr(3+) and Ga(3+) diffusion in periclase: Physics of the Earth and Planetary Interiors, v. 180, no. 3-4, p. 159-171.

Dohmen, R., Becker, H.-W., and Chakraborty, S., 2007, Fe–Mg diffusion in olivine I: experimental determination between 700 and 1,200 C as a function of composition,

crystal orientation and oxygen fugacity: Physics and Chemistry of Minerals, v. 34, no. 6, p. 389-407.
Dohmen, R., and Chakraborty, S., 2007, Fe–Mg diffusion in olivine II: point defect chemistry, change of diffusion mechanisms and a model for calculation of diffusion coefficients in natural olivine: Physics and Chemistry of Minerals, v. 34, no. 6, p. 409-430.
Dohmen, R., Kasemann, S. A., Coogan, L., and Chakraborty, S., 2010, Diffusion of Li in olivine. Part I: experimental observations and a multi species diffusion model: Geochimica et Cosmochimica Acta, v. 74, no. 1, p. 274-292.
Elphick, S. C., Ganguly, J., and Loomis, T. P., 1985, Experimental determination of cation diffusivities in aluminosilicate garnets: Contributions to Mineralogy and Petrology, v. 90, no. 1, p. 36-44.
Ganguly, J., 2010, Cation Diffusion Kinetics in Aluminosilicate Garnets and Geological Applications: Diffusion in Minerals and Melts, v. 72, p. 559-601.
Ganguly, J., Bhattacharya, R., and Chakraborty, S., 1988, Convolution effect in the determination of compositional profiles and diffusion coefficients by microprobe step scans: American Mineralogist, v. 73, p. 901-909.
Ganguly, J., Cheng, W. J., and Chakraborty, S., 1998, Cation diffusion in aluminosilicate garnets: experimental determination in pyrope-almandine diffusion couples: Contributions to Mineralogy and Petrology, v. 131, no. 2-3, p. 171-180.
Jollands, M., O'Neill, H. S. C., Van Orman, J., Berry, A., Hermann, J., Newville, M., and Lanzirotti, A., 2018, Substitution and diffusion of Cr2+ and Cr3+ in synthetic forsterite and natural olivine at 1200–1500° C and 1 bar: Geochimica et cosmochimica acta, v. 220, p. 407-428.
Schindelin, J., Rueden, C. T., Hiner, M. C., and Eliceiri, K. W., 2015, The ImageJ ecosystem: An open platform for biomedical image analysis: Molecular Reproduction and Development, v. 82, no. 7-8, p. 518-529.
Tirone, M., Ganguly, J., Dohmen, R., Langenhorst, F., Hervig, R., and Becker, H.-W., 2005, Rare earth diffusion kinetics in garnet: experimental studies and applications: Geochimica et Cosmochimica Acta, v. 69, no. 9, p. 2385-2398.
Van Orman, J. A., Li, C., and Crispin, K. L., 2009, Aluminum diffusion and Al-vacancy association in periclase: Physics of the Earth and Planetary Interiors, v. 172, no. 1, p. 34-42.
